# Liquid Biopsy in Hepatocellular Carcinoma: Where Are We Now?

**DOI:** 10.3390/cancers13092274

**Published:** 2021-05-10

**Authors:** Filippo Pelizzaro, Romilda Cardin, Barbara Penzo, Elisa Pinto, Alessandro Vitale, Umberto Cillo, Francesco Paolo Russo, Fabio Farinati

**Affiliations:** 1 Gastroenterology Unit, Department of Surgery, Oncology and Gastroenterology, University of Padua, 35128 Padua, Italy; filippo.pelizzaro@unipd.it (F.P.); romilda.cardin@unipd.it (R.C.); barbara.penzo@aopd.veneto.it (B.P.); elisa.pinto@aopd.veneto.it (E.P.); francescopaolo.russo@unipd.it (F.P.R.); 2 Hepatobiliary Surgery and Liver Transplantation Unit, Department of Surgery, Oncology and Gastroenterology, University of Padua, 35128 Padua, Italy; alessandro.vitale@unipd.it (A.V.); cillo@unipd.it (U.C.)

**Keywords:** hepatocellular carcinoma, liquid biopsy, biomarkers, diagnosis, prognosis, extracellular vesicles, circulating nucleic acids, circulating tumor cells

## Abstract

**Simple Summary:**

Hepatocellular carcinoma (HCC) is one of the mostly lethal cancers, with a prognosis which is still very poor. Novel reliable biomarkers, useful in early diagnosis and prognosis assessment, are urgently needed in order to improve HCC patient survival. In recent years, several studies focused on liquid biopsy, the molecular analysis of circulating cancer by-products, as a source of novel biomarkers. Extracellular vesicles, circulating tumor cells, cell-free DNA and non-coding RNA provided very interesting results in a large number of studies published recently, but none of them has entered the clinical routine. In this review we will summarize the available evidence on these novel circulating biomarkers as diagnostic, prognostic, and predictive tools. Liquid biopsy proved to be a very useful source of biomarkers, some of which will probably be applied soon in clinical practice.

**Abstract:**

Hepatocellular carcinoma (HCC) is one of the leading causes of cancer related death worldwide. Diagnostic, prognostic, and predictive biomarkers are urgently needed in order to improve patient survival. Indeed, the most widely used biomarkers, such as alpha-fetoprotein (AFP), have limited accuracy as both diagnostic and prognostic tests. Liver biopsy provides an insight on the biology of the tumor, but it is an invasive procedure, not routinely used, and not representative of the whole neoplasia due to the demonstrated intra-tumoral heterogeneity. In recent years, liquid biopsy, defined as the molecular analysis of cancer by-products, released by the tumor in the bloodstream, emerged as an appealing source of new biomarkers. Several studies focused on evaluating extracellular vesicles, circulating tumor cells, cell-free DNA and non-coding RNA as novel reliable biomarkers. In this review, we aimed to provide a comprehensive overview on the most relevant available evidence on novel circulating biomarkers for early diagnosis, prognostic stratification, and therapeutic monitoring. Liquid biopsy seems to be a very promising instrument and, in the near future, some of these new non-invasive tools will probably change the clinical management of HCC patients.

## 1. Introduction

According to the International Agency for Research on Cancer, in 2018 primary liver tumors ranked as the sixth most common cancer and the fourth leading cause of cancer-related death worldwide [1]. These figures are predicted to increase in the coming decades and it is estimated that more than 1 million people will die due to liver cancer in 2030 [2]. Hepatocellular carcinoma (HCC) account for 85% of all primary hepatic malignancies. The majority of HCC cases occur in patients with underlying liver diseases, mainly due to chronic hepatitis B or C virus (HBV and HCV) infections, alcohol abuse, aflatoxin exposure, or non-alcoholic liver disease (NAFLD) [3]. Despite the recommendation of all available guidelines to apply a regular surveillance in patients at risk, HCC is often diagnosed in advanced stages when curative therapies are no longer feasible. As a consequence, despite the remarkable progresses in therapy, the prognosis of HCC patients remains dismal, with a 5-years survival rate ranging around 20% [4].

Currently, according to guidelines, liver biopsy has a limited role in the management of HCC patients. This is due to the fact that, in patients with liver cirrhosis, a non-invasive diagnosis in the presence of typical imaging features (hypervascularity in the arterial phase and wash-out in portal venous and/or delayed phases) has high specificity. On the other hand, biopsy is indicated for patients without cirrhosis or for cirrhotics with lesions not showing the peculiar and specific radiologic appearance [5]. In most cases liver biopsy, which is associated with a small but still present risk of bleeding and tumor seeding, is unnecessary. Nevertheless, the debate on a more widespread use of liver biopsy is still open [6], with the expansion in recent years of therapeutic possibilities and in consideration of the identification of molecular markers of susceptibility to available systemic treatments, in an attempt of tailoring first and subsequent lines of therapy [7]. However, a high degree of spatial and temporal heterogeneity is present in HCC. Some somatic mutations occur early during tumorigenesis and propagate in many clones, whereas later mutations are present only in some clones (spatial heterogeneity) [8]. Moreover, different therapies select rare mutants and treatment-resistant clones, leading to the development of several genetic backgrounds at different times (temporal heterogeneity) [9,10]. Therefore, a single biopsy is unlikely to represent the entire biology of the tumor, thus limiting the utility of tissue sampling, beyond confirming the diagnosis [11].

**Figure 1:**
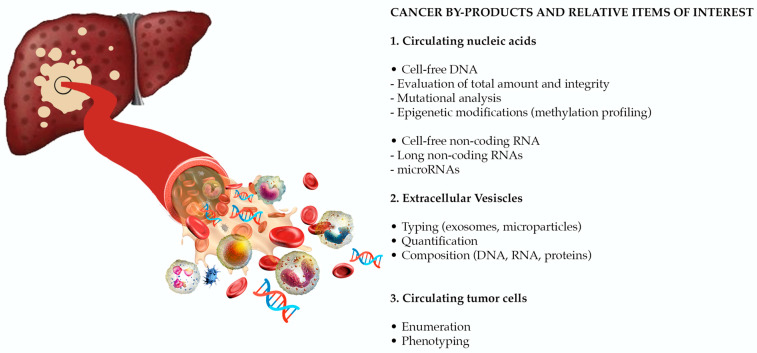
Liquid biopsy is the molecular analysis of cancer by-products released in the bloodstream. Novel potential biomarkers are represented by circulating nucleic acids, extracellular vesicles (EVs), and circulating tumor cells (CTCs). (Adapted from Labgaa et al. [24]).

The European Association for the Study of the Liver (EASL) recognizes as an urgent unmet need the identification of reliable biomarkers, for risk stratification and early HCC detection, prediction of prognosis, and of response to therapy (in particular to systemic treatments) [5]. Despite its unsatisfactory performance in early diagnosis and prognostication [12,13,14,15,16], alpha-fetoprotein (AFP) is still the most widely used biomarker in the clinical management of patients with HCC. Other protein biomarkers, such as des-λ-carboxyprothrombin [17], glypican-3 [18], osteopontin [19], Golgi protein-73 [20], and squamous cell carcinoma antigen [21,22,23] have been evaluated, with erratic results. In the spectrum of circulating molecules derived from the primary tumor (“HCC circulome”), other biomarkers emerged as appealing tools in overcoming the limitations of conventional biomarkers and of tissue biopsy in diagnosis and prognosis. Liquid biopsy is defined as the molecular analysis of circulating cancer by-products, such as extracellular vesicles (EVs), circulating tumor cells (CTCs), and circulating tumor nucleic acids (Figure 1). In recent years, a large evidence has been published, paving the way for the use of liquid biopsy as a source of reliable biomarkers for early tumor detection, prognostic stratification, disease monitoring and evaluation of response to treatment. Considering that these non-invasive biomarkers will probably revolutionize the management of patients with HCC in the near future, with this review we aimed to provide a comprehensive overview of the most relevant available data on the role of liquid biopsy in HCC.

## 2. Circulating Nucleic Acids

Circulating nucleic acids, released in the bloodstream through active secretion or following apoptosis, necrosis or lysis of tumor cells and circulating tumor cells, can be subgrouped in “cell-free DNA” (cfDNA) and “cell-free RNA” (cfRNA). cfDNA can be found in circulation as short nucleosome-associated fragments or long fragments incapsulated in EVs, while cfRNA is usually detected in association with proteins, proteolipid complexes, and EVs due to its relative instability [25].

The analysis of circulating nucleic acids represents a very promising liquid biopsy strategy for getting information on liver tumors. Beyond the utility in risk prediction, early detection, and monitoring treatment response, cfDNA and cfRNA are optimal candidates for tumor molecular profiling. Unlike tumor biopsy, their ability to mirror tumor heterogeneity represents a powerful tool to identify point mutations, aberrant methylation and chromosomal aberrations conferring drug resistance and guiding molecular target therapy [26].

### 2.1. Cell-Free DNA

The original discovery of cfDNA from sera of healthy individuals dates back to 1948. Following the demonstration of high serum concentration of cfDNA in patients with gastrointestinal cancers [27], its potential role as tumor marker emerged when KRAS mutations were identified in cfDNA from patients with colorectal and pancreatic cancers [28,29,30]. From this starting point, a large number of studies has been conducted focusing on the utility of cfDNA analysis also in HCC (Table 1).

#### 2.1.1. Cell-Free DNA Amount and Integrity

The easiest way to use circulating DNA as a biomarker is through the evaluation of its total amount, since a high level of cfDNA in blood reflect cancer growth and tumor burden [31,32,33,34,35,36,37,38]. In 2006, Iizuka et al. [31] demonstrated that cfDNA was able to identify HCC in a cohort of HCV positive patients with a sensitivity of 69.2% and a specificity of 93.3% (AUC = 0.90), both higher than those of AFP. These early results are in line with previous data from our research group: the total amount of cfDNA achieved a sensitivity of 91%, a specificity of 43%, and an AUC of 0.69 in discriminating HCC from CLD and cirrhotic patients [34]. Since cfDNA is not specific for liver cancer, several studies reported an increased diagnostic accuracy when its determination was combined with other biomarkers (i.e., AFP) [32,33,38]. cfDNA have an average size of ~180 base pairs and its fragmentation is a nonrandom process, since liver cfDNA has been found to end at specific genomic coordinates [39]. Interestingly, shorter cfDNA was found in HCC patients compared to non-cancer patients, probably reflecting that not only apoptosis, but also necrosis of tumor cells contributes to the pool of circulating DNA [40,41]. Some researchers demonstrated that the evaluation of length and integrity of cfDNA achieved a diagnostic accuracy comparable to that of AFP [36,42]. The measure of cfDNA total amount or integrity may also be useful as a prognostic biomarker. In their seminal study, Tokuhisa et al. [43] demonstrated that higher levels of cfDNA after liver resection in patients with HCV-related HCC were associated with an increased risk of metastases (adjusted hazard ratio [HR] = 4.5, 95% CI 1.3–14.9) and poorer overall survival (OS) (adjusted HR = 3.4, 95% CI 1.5–7.6). Several other subsequent studies confirmed that patients with high levels of cfDNA had a worse prognosis after different treatments (liver transplantation, liver resection and sorafenib) [34,44,45]. Moreover, a poorer OS was also demonstrated in patients with decreased cfDNA integrity (adjusted HR = 1.86, 95% CI 1.20–2.88) in the study by El-Shazly et al. [36].

When dealing with cfDNA amount as a cancer biomarker, it should be noted that the circulating DNA does not derive only from tumor cells. More precisely, the fraction of cfDNA directly attributable to the presence of cancer is named circulating tumor DNA (ctDNA) [46]. Although patients with cancer have higher cfDNA levels compared to healthy subjects, ctDNA represent a small proportion of the total amount and its level depend on disease burden, stage, cellular turnover and treatment response [47]. Moreover, high quantities of cfDNA are not cancer specific, being also elevated in inflammatory and autoimmune diseases (cirrhosis, chronic hepatitis, systemic lupus erythematous, and rheumatoid arthritis), in pregnancy, and after physical exercise [27,47]. This low specificity may scale back the role of whole cfDNA quantification as diagnostic biomarkers. Nevertheless, a remarkable study demonstrated that the cell and tissue of origin of cfDNA could be inferred by the analysis of the position of nucleosomes [48]. Snyder et al. demonstrated that since nucleosomes, the basic unit of chromatin, are placed in different positions depending on the cell type, nucleosome footprint in cfDNA could be useful to determine the relative contribution of cancer cells to the total circulating DNA pool [48].

#### 2.1.2. Mutations

The majority of studies on cfDNA focused on mutational analysis and epigenetic characteristics, such as its methylation signature. HCC, when compared to other solid tumors, has a lower mutational burden [49]. The main driver somatic mutations affect telomere integrity (TERT promoter, 44%), cell cycle (TP53, 31%), and WNT signaling (CTNNB1, 27%) [50]. Less commonly AXIN1, ARID1A, ARID2, BAP1, RB1, and KEAP1 are mutated (5–10%) [50]. In addition, genetic alterations may be present, including broad chromosome gains and losses with high-level DNA amplifications of chromosomes 6p21 and 11q13, loci corresponding to VEGFA and CCND1/FGF19, respectively [49]. A relevant proportion of the mutations found in HCC biopsies are also detectable in cfDNA (43–83%) [45,51]. According to Howell et al. [52], all the mutations found in the plasma cfDNA matched with tissue mutations, while only 71% of mutations on tumor tissue were found in circulating DNA. When dealing with mutational analysis of cfDNA, we must keep in mind that mutations are more easily identified in advanced disease. In a recent study, at least one mutation in cfDNA was found in almost all (6/7) patients with a tumor ≥5 cm or with metastases, while only 9% of mutations were detected in the cfDNA of patients with smaller, not metastatic HCC [53]. Others reported that, in 48 patients, at least one type of mutation among TP53 (c.747G > T), CTNNB1 (c.121A > G, c.133T > C), or TERT (c.1-124C > T) was documented in 56.3% of patients; only 22.2% of patients had matched mutations in HCC tissue, while none of these mutations were found in non-tumoral liver tissue or in peripheral mononuclear cells [54]. In parallel to what was found in HCC tissue, TP53 is the most commonly mutated gene in cfDNA [55]. In particular, TP53 c.747G > T (p.R249S) mutation appears to be highly specific, since Cohen et al. [56] found it in approximately 20% of HCC blood samples and, conversely, in only 3–4% of pancreatic and stomach cancer samples and in none of more than 800 healthy controls. Although confirming a very high specificity (100%), another study showed a very poor sensitivity (7.6%) for the analysis of TP53 R249S mutation alone in cfDNA [57]. In order to overcome this limitation, the accuracy of TP53 mutation in association with other mutations in a diagnostic panel was evaluated [58,59,60]. Qu et al. demonstrated that a score including several cfDNA mutations (TP53, TERT, CTNNB1 and AXIN1, and HBV integrations), in combination with protein biomarkers (AFP and DCP), age and gender efficiently identified early-stage HCC in a high-risk HBsAg-seropositive population [60]. Sensitivity and specificity, 85% and 93% in the training cohort, were even better in the validation cohort (100% and 94%, respectively) [60]. Moreover, the positivity of TP53 R249S mutation in cfDNA proved to be useful also as prognostic biomarker in a large study involving 895 HCC patients, being a predictor of poorer OS and shorter progression-free survival (PFS) in patients with or without liver resection [61].

The human telomerase reverse transcriptase (TERT) gene encodes for the catalytic subunit of telomerase, which acts together with multiple molecules to maintain telomere homeostasis and chromosomal integrity [62]. The mutations found in TERT promoter lead to TERT reactivation and cell immortalization. Male patients with HCV and/or alcoholic related cirrhosis have a higher prevalence of TERT promoter mutations both in tumor tissue and in cfDNA [63], providing the rationale for TERT promoter mutations analysis in cfDNA for early detection in some populations at risk of developing HCC. In addition, presence of TERT promoter mutation in cfDNA has been associated with poor prognosis after different treatments [58,63,64,65].

#### 2.1.3. Methylation/Epigenetics

Changes in DNA methylation, particularly in the CpG islands of tumor suppressor genes, have been demonstrated to be pivotal in HCC development [66]. Analysis of the methylation pattern of cfDNA may have a value as diagnostic and prognostic biomarker, and might reveal information about tumor size, risk of metastatic spread, and recurrence [67]. Alterations in DNA methylation patterns in HCC tumor tissue after liver resection have been described for many genes. In particular, hypermethylation was found in p15, CDKN2A (encoding for p16), glutathione S-transferase (GSTP1), Ras association domain family 1A (RASSF1A), APC, SOCS1, SOCS3, TIMP3, blood vessel epicardial substance (BVES), and Homeobox A9 (HOXA9) genes, while hypomethylation in long interspersed element-1 (LINE-1) repetitive sequence [67,68,69,70,71,72,73]. However, only a proportion of cfDNA carried the same methylation patterns: hypermethylation of GSTP1 and RASSF1A was found in 50% and in 70–93% of cases respectively, while hypomethylation of LINE-1 in approximately 67% of cases [71,72]. Nevertheless, a large number of studies investigated the diagnostic accuracy of the methylation patterns in several different genes, demonstrating a diagnostic accuracy comparable or even superior to that of AFP. A very high diagnostic accuracy could be obtained with methylation scores, which combine methylation patterns in different genes. Wen et al. [74] demonstrated that a methylation score derived from the analysis of more than 10 genes achieves a sensitivity of 94% and a specificity of 89%. Lu et al. [75] obtained an AUC of 0.87 analyzing the methylation of APC, COX2, RASSF1A, and miR-203, compared to an AUC of 0.56 for AFP. In another study, the methylation of RASSF1A, BVES, and HOXA9 achieved a 73.5% sensitivity and a 91.1%, specificity, with an AUC of 0.83 [70]. A very high diagnostic accuracy in distinguish HCC patients from cirrhotics (sensitivity/specificity 95%/86%, AUC = 0.93) was reported by Kiesel et al. for a score composed by the analysis of HOXA1, EMX1, ECE1, AK055957, PFKP, and CLEC11A methylation in a discovery, phase I pilot and phase II clinical validation cohort study [76]. Cai et al. developed and validated a non-invasive diagnostic model based on Genome-wide mapping of 5-hydroxymethylcytosines in cfDNA achieving an AUC of 0.85 in distinguish early HCC from chronic liver disease (CLD), thus outperforming AFP (AUC = 0.69) [77]. The methylation analysis of cfDNA demonstrated to be useful also in predicting prognosis. RASSF1A methylation was positively correlated with tumor size, while LINE-1 hypomethylation was associated with HCC progression and patients’ survival. The combination of these two genes methylation status was able to predict tumor recurrence after liver resection [71]. The role of LINE-1 hypomethylation in predicting poor prognosis was also confirmed by other researchers [78,79].

In a very interesting recent study including 1098 HCC patients and 835 controls, the authors constructed a diagnostic model with 10 methylation markers in cfDNA, achieving a sensitivity of 85.7% and a specificity of 94.3% in the training cohort (560 normal samples and 715 HCC) [80]. In the validation cohort (275 normal samples and 383 HCC) the model demonstrated a sensitivity of 83.3% and a specificity of 90.5%, thereby differentiating HCC patients from normal controls with an AUC of 0.966 [80]. In the same study, the prognostic score, which was based on the evaluation of the methylation profile of 8 different genes, was associated with higher mortality both in the training (HR = 2.41, 95% CI 1.90–3.03) and in the validation cohort (HR = 1.55, 95% CI 1.25–1.92) [80].

In the chapter of epigenetic biomarkers, nucleosomes and extracellular histones are also emerging. Nucleosomes, beyond being fundamental for genome compaction in the nucleus, may regulate genes expression through their composition and post-translational modifications [81]. Their circulating levels are increased in stroke, trauma, and sepsis [82]. In addition, circulating nucleosome demonstrated a remarkable diagnostic and prognostic performance in several human malignancies, including pancreatic [83], lung [84], colorectal [85], and breast cancers [86]. Moreover, circulating histones have been demonstrated to be key mediators of lethal sepsis [87] and liver inflammatory injury [88]. Some studies demonstrated an involvement of macro histone variants (in particular macroH2A1) in modulating HCC progression and stem cell differentiation [89,90]. There is still poor evidence about circulating nucleosomes and cell-free histones/histone complexes as liquid biopsy biomarkers in HCC. Nevertheless, some interesting results have been achieved in obesity and metabolic fatty liver disease (MAFLD), both risk factors for HCC development. A strong correlation between fatty liver index (a predictor of MAFLD based on BMI, waist circumference, triglycerides, and GGT) and high levels of circulating nucleosomes have been found in obese patients with MAFLD [91]. Moreover, a circulating histone signature (depletion of histone variants macroH2A1.1 and macroH2A1.2, individually or in complex with H2B) identified the severity of steatosis in subjects with lean MAFLD [92]. These encouraging results, together with the simple methodology of the determination (ELISA), could pave the way to the evaluation of circulating nucleosomes and cell-free histones/histone complexes as diagnostic and prognostic biomarkers in HCC.

Overall, a large body of evidence has been produced supporting the great potential of cfDNA as diagnostic and prognostic biomarker in HCC. However, it should be considered that current data largely derive from proof-of-concept retrospective studies, lacking adequate controls (not always including patients at risk of developing HCC, i.e., cirrhotics) and including only a minority of cases with early-stage HCC, which would be candidates for curative treatment options. Moreover, an additional concern regards the lack of standardized protocols for pre-analytical sample preparation, purification, and analysis. Although the use of cfDNA as a liquid biopsy currently presents several limitations in the early detection of HCC, due to the very low amount of cfDNA in the early stages, these approaches may probably dramatically change HCC surveillance. Indeed, a study published more than 10 years ago demonstrated that aberrant methylation of cfDNA fragments was detected up to 9 years before the diagnosis achieved with standard methodology [93].

**Table 1:** Studies on cell-free DNA (cfDNA) as biomarker in HCC patients.

Diagnosis				
Study	cfDNA Property	Number of Patients	Comparator	Main Findings (Sensitivity/Specificity, AUC)
**cfDNA Amount or Integrity**				
Iizuka et al., 2006 [31]	Total amount	52 HCC 30 CLD (HCV) 16 healthy subjects	AFP (cut-off 10.2 ng/mL) DCP (cut-off 29.5 ng/mL)	AFP: 69.2%/72.7% (0.79) DCP: 73.1%/75% (0.73) cfDNA: 69.2%/93.3% (0.90); *p* < 0.05 vs. both AFP and DCP
Ren et al., 2006 [35]	Total amount and chromosome 8p allelic imbalance (D8S258 or D8S264)	79 HCC 20 LC 20 healthy subjects	AFP (cut-off 20 ng/mL)	Total amount of cfDNA: HCC vs. healthy subjects: 52%/95%; 0.80 Allelic imbalance at D8S258 in the plasma of 62% of patients Allelic imbalance at D8S264 in the plasma of 60% of patients High cfDNA concentration + allelic imbalance abnormal in 8/24 patients with low AFP
El-Shazly et al., 2010 [36]	Total amount and integrity	25 HCV-related HCC 25 CLD (HCV) 15 healthy subjects	AFP (cut-off 20 ng/mL)	HCC vs. CLD cfDNA amount: 72%/68%, 0.57 cfDNA integrity: 88%/92%, 0.75
Huang et al., 2012 [32]	Total amount	72 HCC 37 LC or CLD 41 healthy subjects	NR	HCC vs. healthy subjects: 90.3%/90.2%; 0.949 HCC vs. CLD: 59.7%/78.4%; 0.705 cfDNA + AFP (HCC vs. healthy subjects): 95.1%/94.4%; 0.974
Piciocchi et al., 2013 [34]	Total amount	66 HCC 35 LC 41 CLD (HCV)	AFP (cut-off 14 ng/mL)	HCC vs. LC+CLD: cfDNA: 91%/43%; 0.69 AFP: 45%/83%; 0.64
Chen et al., 2013 [33]	Total amount	39 HCC 45 healthy subjects	NR	ctDNA: 56.4%/95.6%; 0.742 AFP: 53.8%/91.1% cfDNA + AFP: 71.8%/86.7% (*p* < 0.05 vs. ctDNA + AFP + AFU group) cfDNA + AFP + AFU: 89.7%/64.4% (*p* < 0.05 vs. ctDNA + AFP)
Huang et al., 2016 [42]	ctDNA integrity	53 HCC 15 benign liver diseases 22 healthy subjects	AFP (cut-off 20 ng/mL)	cfDNA integrity: 43.4%/100%; 0.705 AFP: 50.9%/100%; 0.605 cfDNA integrity + AFP: 79.2%/100%
Marchio et al., 2018 [37]	Total amount, TP53 R249S mutation by digital droplet PCR	149 HCC 164 CLD 49 healthy	AFP (cut-off 10 ng/mL)	cfDNA amount: AUC = 0.585 AFP: AUC = 0.805 Proportion of droplets with TP53 R249S: AUC = 0.827 (*p* > 0.05 vs. AFP)
Yan et al., 2018 [38]	Total amount	24 HCC 62 CLD (HBV)	AFP (cut-off 80.5 ng/mL)	cfDNA amount: 62.5%/93.6%; 0.82 AFP: 47.8%/93.2%; 0.67 cfDNA + AFP + age: 87%/100%; 0.98
**Mutations**				
Igetei et al., 2008 [57]	TP53 R249S mutation	85 HCC 77 healthy subjects	AFP (cut-off 400 ng/mL)	Sensitivity/specificity: 7.6%/100% Patients with HCC and AFP measurements: 16.7% overall, 20% without increased AFP (*p* > 0.05)
Xu et al., 2015 [94]	Copy number variation: gain in 1q, 7q and 19q; loss in 1p, 9q and 14q	31 HCC 8 LC or CLD	AFP (cut-off 10 ng/mL)	Copy number variation score: All HCCs: 83.9%/100% (AUC = 0.95) HCCs ≤ 5 cm: 68.8%/100% Low AFP: 7/10 positive
Liao et al., 2016 [58]	TERT, CTNNB1 or TP53 mutations	41 HCC 10 healthy subjects	AFP (cut-off 20 ng/mL)	Sensitivity 23% and 13% in high vs. low AFP group, respectively (*p* = 0.70) Specificity 90%
An et al., 2019 [95]	ctDNA mutations (139 somatic mutations)	26 HCC 10 LC 10 CLD	NR	cfDNA: AUC = 0.917 Mutation number: AUC = 0.876 cfDNA (cfDNA concentration times variant allele frequency): AUC = 0.871 Maximal variant allele frequency: AUC = 0.802 AFP: AUC = 0.783
Cai et al., 2019 [96]	Fraction of single nucleotide or copy number variants	34 HCC	NR	cfDNA: sensitivity, 100% AFP: sensitivity, 56% AFP-L3: sensitivity, 50% DCP: sensitivity, 82%
Qu et al., 2019 [60]	HCCscreen: mutations in ctDNA (HVB integrations, TP53, CTNNB1, AXIN1 and TERT promoter), AFP, DCP, age and sex	Training: 65 HCC, 70 CLD Validation: 24 HCC, 307 CLD	None	Training cohort (AFP or US positive suspected individuals): 85%/93%, 0.928 Validation cohort (AFP and US negative individuals): 24/331 patients tested positive and eventually 4/24 develop HCC. None of the negative patients develop HCC. Sensitivity/specificity: 100%/94%
Xiong et al., 2019 [59]	Mutations in TP53, ARID1A, FLCN, SETD2, PTEN, BUB1B, CTNNB1, JAK1, AXIN1, EPS15 or CACNA2D4	37 HCC 6 healthy subjects	AFP (cut-off 400 ng/mL)	cfDNA mutations overall: 65%/100%, 0.92 AFP negative: 73%/100%, 0.96 AFP positive: 53%/100%, 0.86
**Methylation/epigenetics**				
Chu et al., 2004 [97]	p16 methylation	46 HCC 23 LC	AFP (cut-off 20 ng/mL)	Overall cohort (sensitivity/specificity): 48%/83% Normal AFP (sensitivity): 44%
Yeo et al., 2005 [98]	RASSF1A methylation	40 HCC 10 healthy subjects	AFP (cut-off 20 ng/mL)	Overall (sensitivity/specificity): 43%/100% Low AFP (sensitivity): 36%
Chan et al., 2008 [99]	RASSF1A methylation	63 HCC 63 CLD (HBV) 50 healthy subjects	AFP (cut-off 20 ng/mL)	RASSF1A methylation detected in: 93% HCC (50% among normal AFP); 58% CLD; 8% healthy subjects
Iizuka et al., 2011 [100]	SPINT2 and SRD5A2 methylation	Training cohort: 108 HCC, 56 CLD Validation cohort:112 HCC, 146 CLD	AFP (cut-off 20 ng/mL) DCP (cut-off 40 mAU/mL)	Methylation of SPINT2 and SRD5A2 + AFP + DCP (sensitivity/specificity): 82.4%/82.1% (training cohort); 73.2%/87.7% (validation cohort) AUC = 0.72 for ≥5 cm HCC and 0.89 for >5 cm HCC AFP alone (sensitivity/specificity): 57.4%/85.7% (training cohort) DCP alone (sensitivity/specificity): 60.2%/89.3% (training cohort)
Sun et al., 2013 [101]	TFPI2 methylation	43 HCC 24 CLD (HBV) 26 healthy subjects	AFP (cut-off 400 μg/L)	TFPI2 methylation (sensitivity/specificity): HCC vs. healthy: 46.5%/80.8% HCC vs. CLD: 46.5%/83.3% AFP alone (sensitivity): 54% TFPI2 + AFP (sensitivity): 61%
Han et al., 2014 [102]	TGR5 promoter methylation	160 HCC 88 CLD (HBV) 45 healthy subjects	AFP (cut-off 20, 200 and 400 ng/mL)	TGR5 methylation frequency: HCC 48%, CLD 14% and healthy subjects 4% HCC vs. CLD (sensitivity/specificity) TGR5 alone: 48.1%/86.4% TGR5 methylation + AFP (200 ng/mL): 68.1%/78.4% AFP (200 ng/mL): 30.6%/92.1%
Huang et al., 2014 [103]	INK4A promoter methylation	66 HCC 43 CLD	AFP (cut-off 200 ng/mL)	INK4A methylation: sensitivity, 74.2% AFP: sensitivity, 45.5% INK4A methylation + AFP: sensitivity, 80.3% (*p* < 0.05 vs. AFP)
Ji et al., 2014 [104]	MT1M and MT1G methylation	121 HCC 37 CLD (HBV) 31 healthy subjects	AFP (cut-off 20 ng/mL)	MT1M or MT1G methylation: HCC vs. CLD: 90.0%/81.1%, 0.86 HCC vs. healthy: 90.9%/83.9%, AUC = NR AFP alone: HCC vs. CLD: 56.0%/62.1%
Kuo et al., 2014 [105]	HOXA9 methylation	40 HCC 34 healthy subjects	AFP (cut-off 10 ng/mL)	HOXA9: 73.3%/97.1%, 0.835 HOXA9 or AFP: 94.6%/97.1%
Li et al., 2014 [106]	IGFBP7 promoter methylation	136 HCC 46 CLD (HBV) 35 healthy subjects	AFP (cut-off 20 ng/mL)	IGFBP7: 65%/83%, 0.740 AFP: 57%/52%, 0.618 IGFBP7 + AFP: 85%/41% (*p* < 0.05 vs. AFP)
Kanekiyo et al., 2015 [107]	RASSF1A, CCND2, CFTR, SPINT2, SRD5A2 and/or BASP1 methylation	125 HCC (HCV)	AFP (cut-off 20 ng/mL) DCP (cut-off 40 ng/mL)	Serum methylation score: Positive in 41% high vs. 48% low AFP Positive in 42% high vs. 46% low DCP (*p* > 0.05 for both)
Wen et al., 2015 [74]	Methylation score: RGS10, ST8SIA6, RUNX2, VIM, CACNA1C, TBX2, SOX9 5’end), NEDD4L intron), ALX3, ZNF683 (3’ end), KCNQ4 (i), ERG, PTPN18 (intron), SYN2, LINC00682 (3’ end), CPLX1 (intron), FLJ42709, UBD (3’ end), SNX10 (3’ end), TRPS1 (intron)	36 HCC 17 CLD 38 healthy subjects	AFP (cut-off 20 ng/mL)	Two cfDNA methylation scores, either score positive (sensitivity/specificity): Training set: 93%/91% Validation set: 100%/80% Combined cohort: 94%/89% Sensitivity 100% in patients with low AFP (*n* = 10)
Dou et al., 2016 [108]	CDH1, DNMT3b or ESR1 promoter methylation	183 HCC 47 LC 126 CLD (HBV) 50 healthy subjects	NR	Methylation frequency: HCC: CDH1 31%, DNMT3b 41%, ESR1 31% CLD: <10% for all 3 genes Healthy subjects: 0% HCC vs. CLD Methylation of any gene (AUC): 0.75; AFP (AUC): 0.63 HCC vs. LC Methylation of any gene (AUC): 0.73; AFP (AUC): 0.62
Hu et al., 2017 [109]	UBE2Q1 hypomethylation	80 HCC 40 LC 40 CLD (HBV) 20 healthy subjects	AFP (cut-off 20, 200 and 400 ng/mL)	UBE2Q1 methylation: 66.3%/57.5%, 0.619 AFP alone: 53.8%/87.5%, 0.668 UBE2Q1 methylation + AFP: 53.8%/87.5%, 0.760
Lu et al., 2017 [75]	Methylation score: APC, COX2, RASSF1A and miR-203	203 HCC 104 CLD 50 healthy subjects	AFP (cut-off 20 ng/mL)	In HBV-related HCC: Methylation score: 84.1%/83.0%, 0.87 AFP: 50.9%/62.1%, 0.56
Xu et al., 2017 [80]	Methylation score: cg10428836, cg26668608, cg25754195, cg05205842, cg11606215, cg24067911, cg18196829, cg23211949, cg17213048, cg25459300	1098 HCC 835 healthy subjects	AFP (cut-off 25 ng/mL)	Training set: 85.7%/94.3%, 0.97 Validation set: 83.3%/90.5%, 0.94 AFP, AUC 0.82 (*p* < 0.05 vs. cfDNA)
Dong et al., 2017 [70]	RASSF1A, APC, BVES, TIMP3, GSTP1, HOXA9 methylation	98 HCC 75 LC 90 CLD (HBV) 80 healthy subjects	AFP (cut-off 20 ng/mL)	HCC vs. CLD RASSF1A, BVES and HOXA9 methylation: 73.5%/91.1%, 0.834 RASSF1A, BVES and HOXA9 methylation + AFP: 83.7%/78.9%, 0.852
Oussalah et al., 2018 [110]	SEPT9 methylation	Derivation cohort: 51 HCC 135 CLD Validation cohort: 47 HCC 56 CLD	NR	Derivation cohort: SEPT9 methylation: 94.1%/84.4%, 0.94 Validation cohort: SEPT9 methylation: 85.1%/87.9%, 0.93 AFP alone (AUC): 0.85 (*p* = 0.002 vs. SEPT9 methylation)
Kisiel et al., 2019 [76]	Methylation score: HOXA1, EMX1, ECE1, AK055957, PFKP, CLEC11A	116 HCC 80 CLD 98 healthy subjects	AFP (cut-off 10 ng/mL)	HCC vs. LC: 95%/86%, AUC 0.93 (no improvement with addition of AFP) HCC vs. healthy: 95%/95% Sensitivity based on cancer stage: 75% (BCLC stage 0), 93% (A/B), 100% (C/D)
Cai et al., 2019 [77]	5-hmC modifications in ctDNA	1204 HCC 392 LC or CLD 958 healthy subjects	AFP (cut-off 20 ng/mL)	Early-stage HCC vs. CLD (AUC): 5-hmC based score: 0.873 (training cohort) and 0.846 (validation cohort) AFP: 0.793 (training cohort) and 0.692 (validation cohort)
**Prognosis**				
**Study**	**cfDNA Property**	**HCC Patients**	**Stage /Treatment**	**Main Findings**
**cfDNA Amount/Integrity**				
Ren et al., 2006 [35]	Total amount and chromosome 8p allelic imbalance (D8S258 or D8S264)	*N* = 79	TNM stage I+II/III+IV: 62%/38% Treatment: NR	Better 3-years DFS associated with low cfDNA (*p* = 0.008), allelic imbalance at D8S258 (*p* = 0.004), allelic imbalance at D8S264 (*p* = 0.01). Better 3-years OS associated with low cfDNA (*p* < 0.0001) and allelic imbalance at D8S258 (*p* = 0.02). AI at D8S258 + higher cfDNA associated with better 3-year DFS (*p* < 0.0001) and 3-year OS (*p* < 0.0001).
Tokuhisa et al., 2007 [43]	Total amount	*N* = 87	TNM stage I/II/III+IV: 46%/44%/10% Treatment: LR	High cfDNA associated with: Poorer OS: HR = 3.4 (1.5–7.6) adjusted for tumor size Higher recurrence in distant organs: HR = 4.5 (1.3–14.9) adjusted for tumor grade Similar DFS (*p* = 0.19)
El-Shazly et al., 2010 [36]	Total amount, integrity	*N* = 25	TNM stage I/II/III/IV: 12%/32%/48%/8% Treatment: NR	OS: cfDNA amount: adjusted HR = 0.54 (0.20–1.60) cfDNA integrity: adjusted HR = 1.86 (1.20–2.88)
Piciocchi et al., 2013 [34]	Total amount	*N* = 66	Stage: 59% Milan in Treatment: NR	Patients with high cfDNA levels showed a significantly shorter OS (24 vs. 37 months; *p* = 0.03). cfDNA was also an independent predictor of survival (HR = NR; *p* = 0.02)
Ono et al., 2015 [45]	Total amount	*N* = 46	Stage: T1/T2/T3/T4 24%/39%/33%/4% (all N0/M0) Treatment: LR or LT	Presence of cfDNA associated with: Increased recurrence (*p* = 0.01) Increased extrahepatic metastases (*p* = 0.04) Similar OS (*p* = 0.07) Increased risk of microscopic vascular invasion: adjusted HR = 6.10 (1.11–33.33)
Park et al., 2018 [111]	Total amount	*N* = 55	TNM stage I/II/III/IV: 23%/23%/27%/27% Treatment: radiotherapy	Higher post-RT cfDNA levels associated with: Similar OS (*p* = 0.15) Similar PFS (*p* = 0.26) Increased hepatic failure: adjusted HR = 2.41 (1.06–5.46) Decreased local control: adjusted HR = 1.96 (0.57–6.81)
Oh et al., 2019 [44]	Total amount, genomic instability and VEGFA amplification	*N* = 151	BCLC stage B/C: 3.3%/96.7%Treatment: sorafenib	Higher amount of cfDNA associated with:Shorter TTP: HR = 1.71 (1.20–2.44), adjusted for AFP Shorter OS: HR = 3.50 (2.36–5.20), adjusted for AFP and MVIGenomic instability associated with: Shorter TTP: HR = 2.09 (1.46–3.00), adjusted for AFP Shorter OS: HR = 3.35 (2.24–5.01), adjusted for AFP and MVI
**Mutations**				
Liao et al., 2016 [58]	TERT, CTNNB1 or TP53 mutations	*N* = 41	Stage: 42% > 5 cm, 27% multiple tumors, 61% vascular invasion Treatment: LR	Presence of mutations associated with: Lower recurrence-free survival (*p* < 0.001); unadjusted analysis only. This was confirmed also in patients with vascular invasion (*p* = 0.003).
Jiao et al., 2018 [63]	TERT mutations	*N* = 218	TNM stage I/II/III+IV: 41.3%/23.4%/35.3% Treatment: NR	Decreased OS in patients with TERT mutations (*p* = 0.006), but not significant association (*p* = 0.19) after adjustment for tumor stage. In patients with HCC on LC, trend toward significance after adjustment for tumor stage (*p* = 0.051)
An et al., 2019 [95]	Any mutation	*N* = 26	TNM stage I/II + III Treatment: LR	Presence of cfDNA post-resection associated with shorter DFS (8.3 months vs. unreached; HR = 7.66, *p* < 0.0001). Improved DFS in patients with high vs. low clearance rate (17.5 vs. 6.7 months; HR = 3.16, *p* = 0.02). Portal vein tumor thrombosis was the other independent prognostic factor.
Cai et al., 2019 [96]	Fraction of single nucleotide or copy number variants	*N* = 34	Stage: NR Treatment: LR	Presence of mutated cfDNA postoperatively: Decrease relapse-free survival (*p* < 0.0001) Decrease OS (*p* < 0.0001) Combination of cfDNA and DCP further increased predictive power
Oversoe et al., 2020 [64]	TERT promoter mutations	*N* = 95	BCLC stage A/B/C/D: 9%/5%/74%/12% Treatment: variable	TERT promoter mutation associated with: Higher mortality: adjusted HR = 2.16 (1.20–3.88). No difference in survival when the analysis was restricted to sorafenib treated patients.
Hirai et al., 2020 [65]	TERT promoter mutations	*N* = 130	TNM stage II + III/IV: 41%/59% Treatment: systemic therapy (66%), TACE (34%)	Presence of TERT promoter mutations associated with: Poorer OS: adjusted HR = 1.94 (1.18–3.24) The worse survival was demonstrated even considering patients treated with systemic therapy and TACE separately
Shen et al., 2020 [61]	TP53 R249S mutation	*N* = 895	TNM stage I + II/III + IV: 67%/33% (cohort 2) Treatment: with (cohort 2) or without (cohort 3) LR	TP53 R249S mutation associated with: Cohort 2 Poorer OS: adjusted HR = 1.79 (1.27–2.52) Poorer PFS: adjusted HR = 1.74 (1.24–2.45) Cohort 3 Poorer OS: adjusted HR = 1.63 (1.30–2.06) Poorer PFS: adjusted HR = 2.03 (1.60–2.59)
Kim et al., 2020 [112]	Total amount and MLH1 single-nucleotide variant	*N* = 107	BCLC stage 0 + A/B + C + D: 48%/52% Treatment: variable	Patients with low cfDNA + MLH1 wild-type had the longest OS, while patients with high cfDNA + MLH1 mutated had the shortest OS.
von Felden et al., 2020 [113]	PI3K/mTOR pathway mutations	*N* = 61	BCLC stage B/C: 30%/70% Treatment: CPI or TKI	Mutations in PI3K/mTOR pathway associated with: Poorer PFS (adjusted *p* = 0.01) in TKI treated patients No association with outcome following CPI
**Methylation/epigenetics**				
Tangkijvanich et al., 2007 [78]	LINE-1 hypomethylation	*N* = 85	CLIP score 0–2/3–5: 48%/52% Treatment: NR	LINE-1 hypomethylation associated with poorer OS: adjusted HR = 1.74 (1.09–2.79)
Huang et al., 2011 [114]	APC or RASSF1A methylation	*N* = 72	TNM stage I + II/III + IV: 24%/76% Treatment: NR	RASSF1A methylation: adjusted HR = 3.26 (1.48–7.21) APC methylation: poorer OS on univariate analysis, but *p* = n.s. after adjustment
Kanekiyo et al., 2015 [107]	RASSF1A, CCND2, CFTR, SPINT2, SRD5A2 and/or BASP1 methylation	*N* = 125	TNM stage I + II/III + IV: 46%/54% Treatment: LR	Methylation of ≥3 genes: Decreased OS: adjusted HR = 2.18 (*p* < 0.001) Decreased DFS: adjusted HR = 4.20 (*p* < 0.001)
Liu et al., 2017 [71]	LINE-1 hypomethylation and RASSF1A promoter hypermethylation	*N* = 75	Stage: 47% ≥ 5 cm (reported only in 49 patients), 16% portal vein thrombosis, 15% lymph node metastases Treatment: LR	LINE-1 hypomethylation associated with: Higher DFS (unadjusted *p* = 0.002) and OS (unadjusted *p* = 0.01) RASSF1A hypermethylation no associated with DFS (*p* = 0.41) and OS (*p* = 0.83) LINE-1 hypomethylation + RASSF1A hypermethylation associated with: Shorter DFS (*p* = 0.0001) and OS (*p* = 0.05). LINE-1 hypomethylation independently associated with poor OS (*p* = 0.045)
Xu et al., 2017 [80]	Methylation of 8 genes: SH3PXD2A, C11orf9, PPFIA1, chromosome 17:78, SERPINB5, NOTCH3, GRHL2, and TMEM8B	*N* = 1049 680 in validation set 39 in training set	TNM stage I/II/III/IV: 16%/16%/52%/12% Treatment: NR	High risk prognostic score associated with poorer OS: Training set: adjusted HR = 2.41 (1.90–3.03) Validation set: adjusted HR = 1.55 (1.25–1.92)
Yeh et al., 2017 [79]	LINE-1 hypomethylation	*N* = 172	BCLC stage 0 + A/B + C: 36%/64% Treatment: NR	LINE-1 hypomethylation was associated with: Shorter OS: adjusted HR = 1.77 (1.12–2.79)
Li et al., 2018 [115]	IGFBP7 promoter methylation	*N* = 155	TNM stage I + II/III + IV: 63%/37% Treatment: LR	Methylation of IGFBP7 associated with: Increased recurrence: adjusted HR = 4.99 (1.51–16.47) Poorer OS: adjusted HR = 3.86 (2.07–7.20)
Chen et al., 2020 [116]	CTCFL hypomethylation	*N* = 43 (+347 HCC from TCGA Atlas)	Stage: 63% size <5 cm, 91% single tumor, 5% metastasesTreatment: NR	CTCFL hypomethylation associated with: Higher risk of postoperative recurrence (*p* = 0.03) Poorer OS (*p* = 0.006)

Abbreviations: AFP, alpha-fetoprotein; AFU, α-L-fucosidase; AUC, area under the curve; BCLC, Barcelona Clinic Liver Cancer; CLD, chronic liver disease; CPI, checkpoint inhibitors; CT, computed tomography; DCP, des-λ-carboxyprothrombin; DFS, disease-free survival; HBV, hepatitis B virus; HCC, hepatocellular carcinoma; HCV, hepatitis C virus; HR, hazard ratio; LC, liver cirrhosis; LR, liver resection; MVI, macroscopic vascular invasion; NR, not reported; OS, overall survival; PFS, progression-free survival; RFA, radiofrequency ablation; TACE, transarterial chemoembilization; TARE, transarterial radioembolization; TCGA, The Cancer Genome Atlas; TKI, tyrosine kinase inhibitors; TNM stage, tumor, nodes, metastases stage; TTP, time to progression; 5-hmC, 2-hydroxymethylcytosine.

### 2.2. Cell-Free Non-Coding RNA

Long and short species of RNA are present in the cell-free non-coding RNA group, both with an extensive involvement in gene expression regulation. The RNA molecules with a length of >200 base pairs are classified as long non-coding RNAs (lncRNAs), several of which are involved in cancer progression. HULC, MEG3, HOTAIR, HOTTIP, MALAT-1, and MVIH are deregulated in HCC, and may be useful as biomarkers [117,118,119,120,121,122,123,124,125,126,127,128]. lncRNA-CTBP, in a panel with other RNA-based biomarkers, showed high sensitivity and specificity in differentiating HCC from cirrhosis and healthy controls [129]. Circulating levels of LINC00152, XLOC014172, and RP11-160H22.5 were able to distinguish HCC patients from cirrhotics, chronic hepatis, and healthy subjects, with very high accuracy when combined with AFP (AUC of 0.986 for HCC vs. chronic hepatitis and 0.985 for HCC vs. healthy controls) [130]. lncRNAs may also be useful as prognostic biomarkers, since they have been shown to predict recurrence after liver transplantation, development of metastases, recurrence-free, and overall survival [120,124,125,126,127,128].

Among short non-coding RNAs, which are generally ~28 base pairs long, microRNAs (miRNAs) are the most extensively studied biomarkers in HCC in recent years, with a role in the diagnosis and in prognosis prediction. miRNAs generally bind to 3’UTR of the target mRNA, resulting in down-regulation of gene expression through translational repression and/or mRNA degradation. More than 70 miRNAs have already been proposed as candidate biomarkers [25]. Table S1 summarizes the most relevant studies on miRNAs as HCC biomarkers.

In the diagnostic setting, highly expressed miRNAs (miR-21, miR-199 and miR-122) seem to be the most promising for the diagnosis of HCC when considered individually, due to their elevated specificity [131]. For instance, Tomimaru et al. [132] demonstrated that miR-21 yielded an AUC of 0.773 with 61.1% sensitivity and 83.3% specificity in differentiating HCC from chronic hepatitis, and an AUC of 0.953 with 87.3% sensitivity and 92.0% specificity in differentiating HCC from healthy volunteers (in both cases superior to AFP). However, in the long run the diagnostic power of a single miRNA turned out to be limited and various panels consisting of more than one circulating miRNA have been evaluated. Lin et al. [133] demonstrated that a seven miRNAs classifier (miR-29a, miR-29c, miR-133a, miR-143, miR-145, miR-192, and miR-505) had a greater AUC compared to AFP in identifying small size and early-stage HCC, detecting also AFP-negative tumors. Another panel consisting of miR-122, miR-192, miR-21, miR-223, miR-26a, miR-27a, and miR-801 was able to distinguish with high accuracy between HCC and healthy controls (AUC = 0.941), chronic hepatitis B (AUC = 0.842), and liver cirrhosis (AUC = 0.884) [134]. Interestingly enough, in a recent study the determination of eight miRNAs showed a sensitivity of 97.7% and a specificity of 94.7% in identifying the presence of HCC among patients at risk, with almost all cancers (98%) diagnosed in early stages [135].

Moreover, circulating miRNAs have a prognostic and predictive significance. Low levels of circulating miR-1, miR-122, miR-26a, miR-29a, and miR-223-3p and high levels of miR-155, miR-96, and miR-193-5p were associated with poor prognosis [136,137,138,139,140,141]. In a recent study, the whole miRNome profile was evaluated in 116 patients with HCC and six miRNAs were identified as prognostic factors; in particular, low levels of miR-424-5p and miR-101-3p and high levels of miR-128, miR-139-5p, miR-382-5p, and miR-410 were associated with lower survival rates [142]. After surgical resection, miR-224 and miR-500 levels decreased [143,144], miR-148a was up-regulated [145], and increased levels of serum miR-1246 could predict early tumor recurrence (<12 months) [146]. High expression of miR-122 as well as low levels of miR-26a and miR-29a have been found to be poor prognostic markers in patients undergoing radiofrequency ablation [138,147] and some authors found that miRNAs evaluation could predict response to TACE [148,149] or sorafenib [150,151]. Recently, a study evaluating plasma samples from participants to the registrative trial of regorafenib (RESORCE) identified 9 plasma miRNAs (miR-30a, miR-122, miR-125b, miR-200a, miR-374b, miR-15b, miR-107, miR-320, and miR-645) whose levels were significantly associated with OS [152].

## 3. Extracellular Vesicles

Extracellular vesicles (EVs) are small membrane vesicles released by cells in extracellular environment in normal physiology and in pathological conditions [153]. EVs transport a variety of bioactive molecules, including mRNA, miRNAs, proteins, and lipids, that can be transferred among cells both in the environment in which they are released, as well as at distant sites, regulating various cell responses [153,154]. Considered their ability of altering intracellular pathways [155,156,157,158], cancer cells can use EVs to take advantage in proliferation [159].

EVs are generally classified in small (exosomes) and large EVs (ectosomes, also called microparticles (MPs) or microvesicles) [160]. Although small and large EVs may be distinguished by some of the expressed markers, such as CD63, HSP70, CD9, CD81, and integrins [161,162], the border between these two entities is not sharp [25]. The growing number of studies providing evidence for a key pathophysiological role of EVs in various aspects of liver diseases and the fact that EVs are released in the systemic circulation, where they are remarkably stable, provide the background to consider their assessment and quantification in blood as a novel form of liquid liver biopsy [66]. Several studies demonstrated a potential role of EVs as biomarkers in HCC patients (Table 2).

First reports showed that HCC patients had a higher level of circulating EVs compared to controls [163] and the determination of total amount of EVs provided slightly better sensitivity and specificity compared to alpha-fetoprotein (AFP) in HCC diagnosis [164]. A specific form of large EVs expressing surface AnnexinV, EpCAM, ASGPR1, and CD133 was identified by Julich-Haertel et al. [165] as a marker able to distinguish HCC and cholangiocarcinoma from other cancer types, cancer-free cirrhotic patients, and healthy subjects. Sensitivity, positive predictive value, and area under the curve (AUC) in the distinction between HCC and cirrhosis were 80%, 73%, and 0.744, respectively [165].

Going beyond the simple determination of the total amount of EVs, the researchers subsequently focused on analyzing their content. Arbelaiz et al. [166] demonstrated that galectin-3-binding protein (LG3BP) and polymeric immune receptor (PIGR) had higher diagnostic accuracy (AUC of 0.904 and 0.837, respectively) compared to AFP (AUC = 0.802). Other promising molecules are exosomal AFP and GPC3 mRNA [167], hnRNPH1 mRNA [168], and long non-coding RNAs (lncRNAs) [169,170,171,172]. In particular, Xu et al. [170] obtained AUCs of 0.894 and 0.885 in derivation and validation cohorts, respectively, with the combination of two lncRNAs (ENSG00000258332.1 and LINC00635). In another study, a machine learning based score (“HCC classifier”) with 8 lncRNAs markers showed very promising AUCs (0.953 in training cohort, 0.983 in validation cohort and 0.963 in testing cohort) [171]. Several other researchers focused their attention on exosomal miRNAs [173,174,175,176,177]. Some studies found similar diagnostic accuracies for AFP and EVs miRNAs [173,175], while others demonstrated the superiority of the latter [174,176].

A lower number of studies investigating EVs in the prognostic field are available, and most of them focused on the evaluation of exosomal miRNAs, in particular after surgical therapies (liver resection or liver transplantation) [177,178,179,180,181,182,183]. The only miRNA included in more than one study was miR-21, and its high levels have been repetitively associated with increased risk of disease progression and poorer survival [182,184,185]. Other studies demonstrated that low levels of exosomal miR-718, miR-125b, miR-638 and miR-320a [177,178,179,181] and high exosomal miR-665 and miR-10b [180,182] were associated with worse prognosis.

EVs and their content are promising candidate biomarkers in patients with HCC for diagnosis and prognosis prediction. Nevertheless, additional larger prospective studies should be conducted to definitely establish their role as liquid biopsy.

**Table 2:** Studies on extracellular vesicles (EVs) as biomarkers in HCC patients.

Diagnosis				
FStudy	EVs Property	Number of Patients	Comparator	Main Findings (Sensitivity/Specificity, AUC)
Wang et al., 2013 [164]	Total amount	55 HCC; 40 LC; 21 healthy subjects	AFP (cut-off 20 ng/mL)	Sensitivity/specificity: 88.9%/62.6% for EVs and 85.7%/40.0% for AFP TNM stage I vs. cirrhosis: AUC = 0.83 (*p* < 0.01 vs. AFP) TNM stage II vs. cirrhosis: AUC = 0.94 (*p* < 0.01 vs. AFP)
Cheng et al., 2015 [163]	Total amount	12 HCC; 11 CLD; 6 healthy subjects	NR	EVs concentration higher in HCC patients vs. healthy controls or cirrhotics. No differences in EVs concentration based on AFP levels.
Julich-Haertel et al., 2017 [165]	Tumor-associated MPs	Explorative study: 22 HCC, 26 CCA, 5 LC, 18 IH, 53 CLD, 18 controls. Validation study: 86 HCC, 38 CCA, 49 LC, 10 NSCLC, 19 CRC, 26 IH, 173 CLD, 58 controls.	NR	Explorative study. HCC vs. controls AnnexinV +, EpCAM + taMPs: 81.8%/66.7%, 0.833 AnnexinV +, EpCAM +, CD147 + taMPs: 72.7%/82.3%, 0.739 Validation study. HCC vs. controls AnnexinV +, EpCAM + taMPs: 76.5%/63.3%, 0.769 AnnexinV +, EpCAM +, CD133 + taMPs: 69.8%/41.4%, 0.626 AnnexinV +, EpCAM +, ASGPR1 +, CD133 + taMPs: 80.0%/50.0%, 0.744 Validation study. Cirrhosis vs. HCC AnnexinV +, EpCAM +, ASGPR1 + taMPs: 81.4%/46.9%, 0.732
Arbelaiz et al., 2017 [166]	EV proteins (LG3BP and PIGR)	29 HCC; 43 CCA; 30 PSC; 32 healthy subjects	AFP	HCC vs. controls LG3BP: 96.6%/71.8%, 0.904 PIGR: 82.8%/71.8%, 0.837 AFP: 82.1%/64.0%, 0.802
Abd El Gwad et al., 2018 [169]	lncRNA-RP11-513I15.6, miR-1262 and RAB11A	60 HCC; 42 CLD; 18 healthy subjects	NR	96.7%/95.0% for lncRNA-RP11-513I15.6 95.0%/80.0% for miR-1262 75.0%/73.3% for RAB11A mRNA 100.0%/76.7% for lncRNA-RP11-513I15.6 + miR-1262 + AFP
Pu et al., 2018 [173]	miR-21-5p and miR-144-3p	24 HCC; 16 CLD; 17 healthy subjects	NR	miR-21-5p: AUC = 0.442 miR-144-3p: AUC = 0.747 miR-144.3p/miR-21-5p ratio: AUC = 0.780 AFP: AUC = 0.626
Wang et al., 2018 [167]	AFP and GPC3 mRNA	40 HCC; 38 healthy subjects	AFP (cut-off 20 ng/mL)	EV AFP mRNA: AUC = 0.947 EV GPC3 mRNA: AUC = 0.979 AFP protein: AUC = 0.936 AFP and GPC3 mRNA: AUC = 0.995
Wang et al., 2018 [174]	miR-122, miR-148a and miR-1246	68 HCC; 53 LC; 50 CLD; 64 healthy subjects	AFP	Cirrhosis vs. HCC (all stages). AUC: miR-122: AUC = 0.816 miR-148a: AUC = 0.891 miR-1246: AUC = 0.785 AFP: AUC = 0.712 miR-122 + miR-148a + AFP: AUC = 0.931
Xu et al., 2018 [170]	lncRNAs (ENSG00000258332.1 and LINC00635)	60 HCC (+55 in validation cohort); 85 LC; 96 CLD (+60 in validation cohort); 60 healthy subjects (+60 in validation cohort)	AFP (cut-off 20 μg/L)	HCC vs. CLD First cohort: ENSG00000258332.1: 71.6%/83.4%, 0.719 LINC00635: 76.2%/77.7%, 0.750 AFP: 54.7%/75.3%, 0.666 All 3 markers: 83.6%/87.7%, 0.894 Second cohort: ENSG00000258332.1: 73.5%/80.5%, 0.718 LINC00635: 79.6%/75.2%, 0.731 AFP: 52.5%/74.1%, 0.634 All 3 markers: 84.5%/85.3%, 0.885
Xu et al., 2018 [168]	hnRNPH1 mRNA	88 HCC; 67 LC; 68 CLD; 68 healthy subjects	AFP (cut-off 20 ng/mL)	HCC vs. CLD hnRNPH1 mRNA: 85.2%/76.5%, 0.865 AFP: 69.3%/87.9%, 0.785 hnRNPH1 + AFP: 87.5%/84.8%, 0.891 HCC vs. cirrhosis hnRNPH1 mRNA: 86.4%/54.0%, 0.647 AFP: 46.6%/88.3%, 0.674 hnRNPH1 + AFP: 50.3%/91.0%, 0.749
Zhang et al., 2019 [175]	miR-212	78 HCC; 95 LC; 58 CLD; 70 healthy subjects	NR	HBV-related HCC vs. healthy subjects miR-212: 70.0%/95.0%, 0.89 AFP: 0.85 Non-HBV-related HCC vs. healthy subjects miR-212: 89.0%/62.0%, 0.79 AFP: 0.84
Li et al., 2019 [171]	lncRNAs	71 HCC; 37 CLD; 94 healthy subjects	AFP (cut-off 10 ng/mL)	Support vector machine model (HCC classifier with 8 markers) Training cohort: 84%/94%, 0.953 Validation cohort: 89%/91%, 0.983 Testing cohort: 85%/95%, 0.963
Lu et al., 2020 [172]	lncRNAs: ENSG00000248932.1 ENST00000440688.1 ENST00000457302.2	200 HCC; 200 CLD; 200 healthy controls	NR	Three lncRNAs: AUC = 0.96/0.53 in training/validation cohorts Three lncRNAs + AFP: AUC = 0.97/0.87 in training/validation cohorts
Sorop et al., 2020 [176]	miR-21-5p and miR-92a-3p	48 HCC; 38 LC; 20 healthy subjects	AFP	AFP alone: AUC = 0.72 miR-21-5p + miR-92a-3p + AFP: AUC = 0.85 (*p* < 0.05 vs. AFP)
Hao et al., 2020 [177]	miR-320a	104 HCC; 55 CLD; 50 healthy subjects	NR	HCC vs. healthy subjects: 77.9%/80.0%, 0.86 HCC vs. CLD: 76.1%/81.8%, 0.83
**Prognosis**				
**Study**	**EVs Property**	**Number of Patients**	**Stage/Treatment**	**Main Findings**
Sugimachi et al., 2015 [178]	miR-718 and miR-1246	*N* = 66 (6 in exploratory and 59 in validation analysis)	Stage: 34% beyond Milan criteria Treatment: LT	Recurrence post-LT: 6/42 in the low and 0/11 in the high miR-718 groups (*p* = n.s.). Patients with tumor diameter ≥3 cm: greater recurrence with high miR-718 (*p* = 0.0002). No association with recurrence for miR-1246
Liu et al., 2017 [179]	miR-125b	*N* = 128	TNM stage I/II–III: 37.5%/62.5% Treatment: LR	Low miR-125b associated with: Lower time-to-recurrence: HR = 0.14 (0.08–0.27); *p* < 0.001 Poorer OS: HR = 0.33 (0.18–0.62); *p* < 0.001
Qu et al., 2017 [180]	miR-665	*N* = 30	TNM stage I–II/III–IV: 20%/80% Treatment: LR	Patients with high miR-665 showed lower OS (*p* < 0.05; HR not reported)
Shi et al., 2018 [181]	miR-638	*N* = 126	TNM stage I + II/III + IV: 53%/47% Treatment: LR	Low miR-638 levels associated with: Poorer OS (adjusted HR = 2.80, 1.24–4.31; *p* = 0.01)
Suehiro et al., 2018 [184]	miR-122 and miR-21	*N* = 75 (57 with LC)	Stage: NR Treatment: TACE	miR-21 and miR-122 not associated with survival in the entire cohort.In LC group, high miR-122 ratio (after/before TACE) associated with poorer OS: adjusted HR = 2.72 (1.04–8.02); *p* = 0.04
Abd El Gwad et al., 2018 [169]	RAB11A mRNA	*N* = 60	BCLC stage early: 90% Treatment: NR	Low levels of RAB11A mRNA are associated with longer recurrence-free survival: adjusted HR = 0.36 (0.15–0.88), *p* = 0.03
Lee et al., 2019 [185]	miR-21 and lncRNA-ATB	*N* = 79	TNM stage I–II/III–IV:40.5%/59.5% Treatment: 10 LR, 5 LT, 24 ABL, 9 TACE, 17 SOR and 14 BSC	High miR-21 and lncRNA-ATB independent predictors of mortality (HR = 2.87 and 2.17, respectively; all *p* < 0.05). High miR-21 and lncRNA-ATB independent predictors of disease progression (HR = 2.53 and 2.55, respectively; all *p* < 0.05).
Tian et al., 2019 [182]	miR-21 and miR-10b	*N* = 124	Stage: 79% monofocal, 35% ≤ 3 cm Treatment: LR	Poorer disease-free survival with: High miR-21: adjusted HR = 2.45 (1.25–4.78); *p* = 0.009 High miR-10b: adjusted HR = 2.55 (1.30–4.99); *p* = 0.006
Hao et al., 2020 [177]	miR-320a	*N* = 104	TNM stage: 37.5%/62.5% Treatment: LR (+/− adjuvant chemotherapy)	Low miR-320a associated with poorer OS and DFS. Low miR-320a independent predictor of mortality: adjusted HR = 2.97 (1.56–4.63); *p* = 0.008
Luo et al., 2020 [183]	circAKT3	*N* = 124	TNM stage I–II/III–IV: 44%/37% Treatment LT/LR: 19/81%	Patients with high circAKT3 have: Higher tumor recurrence rates (HR 3.14, 1.29–6.21; *p* = 0.01) Higher mortality (HR 1.89, 1.04–3.01; *p* = 0.048)

Abbreviations: AFP, alpha-fetoprotein; AUC, area under the curve; BCLC, Barcelona Clinic Liver Cancer; CCA, cholangiocarcinoma; CLD, chronic liver disease; CRC, colorectal carcinoma; DFS, disease-free survival; EVs, extracellular vesicles; HCC, hepatocellular carcinoma; HR, hazard ratio; IH, inguinal hernia; LC, liver cirrhosis; lncRNA, long non-coding RNA; LR, liver resection; LT, liver transplantation; miR, microRNA; MPs, microparticles; NSCLC, non-small cell lung carcinoma; NR, not reported; OS, overall survival; PSC, primary sclerosing cholangitis; TACE, transarterial chemoembolization; taMPs, tumor-associated microparticles; TNM stage, tumor, nodes, metastases stage.

## 4. Circulating Tumor Cells

Metastatization is a complex and largely unknown process requiring the ability for cancer cells to escape from the primary tumor, survive in the circulation, and then settle in a new organ. Circulating tumor cells (CTCs) are key players in cancer dissemination. Considering that CTCs are present in the order of one per billion of blood cells in patients with metastatic disease, there have been some initial obstacles in their study [186]. Nevertheless, technical and methodological advances in the last years led to a significant expansion of publications aimed at investigating their role as candidate biomarkers (Table 3).

Platforms for the detection of CTCs are based on their known biological and physical properties, and can grossly be divided in immunoaffinity-based and biophysical property-based enrichment [187]. Immunoaffinity-based CTCs enrichment techniques use antibodies against cell surface markers bounded to the device or a magnetic substance. The enrichment can be positive when CTCs are captured with antibodies against tumor specific antigens expressed on CTC surface, or negative when hematopoietic cells in the background are depleted by using antibodies against CD45 [188]. The CellSearch™ system (Veridex) captures CTCs using an immunomagnetic separation system with antibodies against EpCAM and cytokeratin coated onto ferrofluid beads and has been approved by the US Food and Drug Administration for use in patients with breast, prostate and colorectal cancers [189,190]. Other developed detection systems include CTC-Chip™ [191], CTC-iChip™ [192], and NanoVelcro™ [193]. These methods rely on tumor expression of the target proteins and their role is limited for cancers that do not typically express them. Only about one third of CTCs in HCC are positive for EpCAM and cytokeratin [194,195], and even if CellSearch™ became the most popular detection system, it could be of limited application in HCC. Moreover, given that epithelial markers such as EpCAM are often downregulated or lost during epithelial-to-mesenchymal transition (EMT) [196], CTC with EMT phenotype which have strong metastatic potential could not be detected by positive enrichment methods that target epithelial markers. Therefore, strategies targeting stem cell markers (CD133), mesenchymal markers (vimentin), and cancer specific antigens (such as HER2, PSMA, ASGPR, Hepar 1, and carbamoyl phosphate synthetase 1) have been developed [197,198]. The biophysical methods to isolate CTCs rely on their typical features such as large size, mechanical plasticity, and dielectric mobility properties, employing centrifugation and filters or flow devices with channels of varying size or nature. Although the advantage of avoiding the challenges of targeting numerous tumor specific epitopes, these methods may be less cancer-specific.

As far as the diagnostic value of CTCs analysis is considered, published studies showed that CTCs may have a role in differentiating HCC from controls. A major concern when dealing with CTCs analysis as diagnostic biomarker is the fact that, since their levels correlate with tumor burden [199], the sensitivity in early-stage disease may be too low. Nevertheless, Guo et al. [200] in a large study investigating a CTC-derived PCR score (quantifying the expression of cancer-related genes in blood), demonstrated a sensitivity of 72.5%, a specificity of 95%, and an AUC of 0.88 (compared with 57%, 90%, and 0.77 of AFP at a cut-off of 20 ng/mL). In addition, this score performed well also in patients with early-stage HCC (AUC of 0.92 in BCLC stage 0 and 0.86 in BCLC stage A).

CTCs evaluation combined with AFP provided incremental performance with respect to AFP alone in identifying HCC patients. In a study, the AUC in the discrimination of CLD and HCC patients was 0.67 for AFP (cut-off 400 ng/mL), 0.77 for CTCs (detected with CanPatrol™), and 0.82 for the combination of both [201]. Guo et al. reported that CTCs, defined by the expression of EpCAM mRNA, had a sensitivity of 42.6% and an AUC of 0.70 in discriminating HCC from CLD and healthy controls, while AFP (cut-off 400 ng/mL) demonstrated a lower sensitivity (39.5%; AUC not reported); the combination of CTCs and AFP increased sensitivity to 73% and the AUC to 0.86 [202].

Considering that CTCs are extremely rare in the circulation and that their number tends to be proportional to tumor volume, which make their detection in early-stage disease challenging [199], they are probably more useful for prognostication rather than for early diagnosis. Indeed, several evidences emerged linking CTCs enumeration with prognosis of HCC patients. A landmark study in 2004 demonstrated that the presence and number of CTCs, identified and enumerated based on their morphology, were associated with shorter survival [203]. Subsequent studies using CellSearch™ showed that the detection of EpCAM positive CTCs was associated with an higher tumor recurrence rate after liver resection [204] and with a worse prognosis [205,206]. The independent prognostic value of CTCs amount was confirmed even with other CTCs enrichment technologies, such as ImageStream flow cytometry, which uses a panel of markers and generates high resolution images of isolated CTCs [195,207]. Beyond simple enumeration, other reports have investigated the prognostic impact of subgroups of CTCs, divided according to cell surface markers, RNA expression, or genomic aberrations. The identification of CTCs with cancer stem cell-like or mesenchymal surface markers is useful to predict tumor recurrence [208,209,210,211]. Other studies demonstrated that CTCs with detectable AFP mRNA were associated with a higher risk of metastatic dissemination [212], whereas CTCs with aneuploid chromosome 8 predicted shorter survival in patients treated with surgical resection [213]. The interesting study by Ha et al. [214] introduced the concept of ΔCTC, which is defined as the variation in CTCs enumeration after surgery, and is an independent factor of lower survival and recurrence after hepatectomy.

Cancer cell dissemination seems to be promoted by treatment, in particular surgical therapies. Liver manipulation is associated with a release of CTCs [215] and the anterior as compared to the conventional surgical approach is associated with a lower release of CTCs as well as better survival [216]. In liver transplantation for HCC, five steps to minimize CTCs dissemination and thereby reduce the risk of recurrence have been described [217]. This approach in transplantation assumes even more importance as an association between CTCs detection and recurrence after transplantation has been demonstrated [218,219]. Overall, data consistently reported that the number of CTCs is a surrogate of poor prognosis, predicting higher recurrence and lower survival. A recent metanalysis and data from experimental models led to the same conclusions [220,221].

Considered that CTCs detection methods are costly and time consuming, the application of CTCs enumeration in clinical practice requires a clear advantage to be established. Probably, this is an unrealistic goal and therefore phenotypic characterization of CTCs may be more useful, since tissue-based biomarkers that could be of help in treatment choice and monitoring are currently lacking. Moreover, it is clear from several studies that CTCs are a heterogeneous population and that they may reflect tumoral heterogeneity better than a tissue biopsy [187,195]. The CTC pERK/pAkt phenotype has recently been reported to predict sensitivity to sorafenib [222], while the presence of CTCs positive for PD-L1 is associated with response to checkpoint inhibitors [223]. Considered that result, it could be imagined that phenotyped CTCs will be useful surrogates for guiding enrichment trials with molecular targeted therapies. Moreover, methods for collecting living CTCs from HCC patients and culture them into three-dimensions spheroid-like structures have also been developed, with the possibility to bring personalized medicine to a new level. In this scenario, Zhang et al. [224] explored individual sensitivity to sorafenib and oxaliplatin after collecting and culturing CTCs, and the evaluation of multiple therapeutic candidates in patients’ CTC-derived xenografts may become a future reality [66].

Even if the use of CTCs analyses as biomarkers in guiding clinical decisions has huge potential, perhaps the most innovative and relevant contribution of CTC studies will be in advancing our understanding of the biology of metastatic disease as well as the development of treatment strategies. The analysis of CTCs at a molecular level, facilitated by the advancements in sequencing technologies, may lead to the identification of new mutations responsible for tumor metastatization and resistance to drugs [225]. Moreover, other insights in metastatic spread have been achieved analyzing the spatial distribution of CTCs in the bloodstream. An interesting study analyzed and compared CTCs collected in HCC patients from different vessels (peripheral veins and arteries, portal vein, and hepatic veins). The greatest number of CTCs was demonstrated in hepatic veins, with a dramatic reduction in peripheral vessels after passage through the lungs. Moreover, there was a phenotypic heterogeneity in CTCs isolated from different sites, being predominantly epithelial into the hepatic vein and EMT-transformed when isolated in peripheral vessels [226]. The CTC burden and the presence of CTC clusters in both hepatic and peripheral veins predicted lung and liver metastases.

Although the rapid evolution in technologies supporting CTCs detection, isolation and characterization and the very promising results in the studies so far published, the clinical application of CTCs as biomarkers is hindered by the different methodologies applied by single researchers. Indeed, few studies have been reproduced by more than one research group. Before the incorporation of CTCs evaluation in trials and clinical practice, standardized protocols with reproducible results, currently lacking in HCC, are needed.

**Table 3:** Studies on use of circulating tumor cells (CTCs) as biomarkers in HCC patients.

Diagnosis				
Study	CTCs Definition	Number of Patients	Comparator	Main Findings (Sensitivity/Specificity, AUC)
Yao et al., 2005 [227]	CD45 (−) EpCAM (+) then AFP mRNA	49 HCC 36 CLD or LC 18 healthy subjects	AFP (cut-off 20 ng/mL)	AFP mRNA (sensitivity/specificity): 72.1%/66.7% Low AFP: sensitivity, 75% High AFP: sensitivity, 71% (*p* > 0.05)
Guo et al., 2007 [228]	CD45 (−) and EpCAM (+), then AFP mRNA	44 HCC 7 healthy subjects	AFP (20 ng/mL)	AFP mRNA (sensitivity): 72.7%; 50% in patients with AFP < 20 ng/mL and 86.7% in patients with AFP >1000 ng/mL (*p* < 0.05)
Xu et al., 2011 [229]	ASGPR (+)	85 HCC 37 CLD or benign liver diseases 20 healthy subjects	AFP (cut-off 20 or 100 ng/mL)	CTCs (sensitivity/specificity): 81%/100% No significant differences in CTCs level according to AFP values
Liu et al., 2013 [210]	CD45 (−) and ICAM-1 (+)	60 HCC	AFP (cut-off 20 ng/mL)	High levels of CTCs in 83.3% of AFP + and 16.7% of AFP negative patients (*p* = 0.14)
Sun et al., 2013 [204]	CellSearch™	123 HCC 5 CLD 10 healthy subjects	AFP (cut-off 400 ng/mL)	≥2 CTCs/7.5 mL: Overall (sensitivity/specificity): 41.5%/100% High AFP: sensitivity, 54.7% Low AFP: sensitivity, 31.4% (*p* = 0.009)
Bahnassy et al., 2014 [230]	CD45 (−) and either CK19, CD90 or CD133 (+)	70 HCC 30 CLD (HCV) 33 healthy subjects	AFP ratio (undefined)	CTCs have poorer performances compared to AFP. HCC vs. CLD: AFP ratio: 95.7%/90.5%, 0.99 CK19 (+) CTCs: 87.1%/82.5% CD90 (+) CTCs: 81%/89.6% CD133 (+) CTCs: 40%/6.3%
Fang et al., 2014 [231]	CellSearch™	42 HCC 10 CLD 10 healthy subjects	AFP (cut-off 40 ng/mL)	CTCs (sensitivity/specificity): 74%/100% Sensitivity 89% among patients with high AFP and 61% among those with low AFP (*p* = 0.08)
Guo et al., 2014 [202] ^†^	CellSearch™ and quantitative PCR for EpCAM in CD45 (−) cells	122 HCC 25 CLD or LC (HBV) 24 benign tumors 71 healthy subjects	AFP (cut-off NR)	HCC vs. other groups: EpCAM-mRNA (+) CTCs: 42.6%/96.7%, 0.70 EpCAM-mRNA (+) CTCs + AFP: 73%/93.4%, 0.86
Kelley et al., 2015 [194]	CellSearch™	20 HCC 10 CLD	AFP (400 ng/mL)	CTC detection in 7 of 20 (35%) HCC patients and 0 of 9 CLD (*p* = 0.04). AFP ≥ 400 ng/mL: sensitivity 70% AFP < 400 ng/mL: sensitivity 10% (*p* = 0.008)
Zhou et al., 2016 [232]	CD45 (−) EpCAM-mRNA (+)	49 HCC	AFP (cut-off 400 ng/mL)	Any CTCs (sensitivity): Overall: 34.6% Low vs. high AFP: 28.2% vs. 60% (*p* = 0.06)
Kalinich et al., 2017 [233]	PCR assay: AFP, AHSG, ALB, APOH, FABP1, FGB, FGG, GPC3, RBP and TF	63 HCC 31 CLD 26 healthy subjects	AFP (cut-off 100 ng/mL)	PCR score +: 9 of 16 (56%) untreated HCC patients, 1 of 31 (3%) CLD and 2 of 26 (7.6%) healthy subjects. 15 patients with both PCR score and AFP available: 4 (27%) PCR score +, 1 (7%) AFP +, 5 (33%) PCR score + and AFP + 6 patients within Milan criteria: 2 (33%) PCR score + and 0 (0%) AFP +
Bhan et al., 2018 [234]	CD45 (−) and hydrodynamics, followed by HCC score based on gene expression	54 HCC 39 CLD 10 healthy subjects	AFP (cut-off 20 ng/mL)	HCC score outperformed AFP in identifying HCC vs. CLD (sensitivity/specificity): HCC score: 85%/95% AFP: 55%/100%
Guo et al., 2018 [200] ^†^	CTC detection panel: PCR for EpCAM, CD133, CD90 and CK19	Training and validation cohorts: 395 HCC 301 CLD and LC (HBV) 210 healthy subjects	AFP (cut-off 20 ng/mL)	Validation cohort (sensitivity/specificity, AUC): HCC vs. other groups: CTC detection panel: 72.5%/95%, 0.88 AFP: 57%/90%, 0.77 CTC detection panel + AFP: 76%/95%, 0.89 Early-stage HCC vs. other groups: CTC detection panel: 71.8%/95%, 0.87 AFP: 53.4%/90%, 0.74 CTC detection panel + AFP: 80.9%/87%, 0.88 AUC in different stages: 0.92 (BCLC 0), 0.86 (BCLC A), 0.91 (BCLC B), 0.86 (BCLC C) In AFP negative patients: 77.7%/95%, 0.89
Xue et al., 2018 [235]	CellSearch™ and iFISH (either CD45 (−) CK (+) DAPI (+) and hybridization signal for CEP8 ≥2 or CD45 (−) CK (−) DAPI (+) and hybridization signal for CEP8 > 2)	30 HCC 10 healthy subjects	AFP (400 IU/mL)	CTCs measured by CellSearch™ (sensitivity/specificity): 26.7%/100% CTCs measured by iFISH (sensitivity/specificity): 70/100% Low AFP: sensitivity, 90% High sensitivity, 30% (*p* = 0.002)
Yin et al., 2018 [236]	CanPatrol™	80 HCC 10 healthy subjects	AFP (cut-off 20 ng/mL)	Overall cohort (sensitivity/specificity): Any CTCs: 77.5%/100% Twist (+) CTCs: 67.5%/100%Low AFP: sensitivity, 35.3% or 17.7% for any CTCs or Twist (+) CTCs, respectively (*p* < 0.001) High AFP: sensitivity, 88.9% or 71.8%for any CTCs or Twist (+) CTCs, respectively (*p* < 0.001)
Cheng et al., 2019 [201]	CanPatrol™	113 HCC 57 benign liver lesions	AFP (cut-off 400 μg/L)	CTCs outperformed and provided incremental benefit to AFP.AFP: 44.3%/89.5%, 0.67 Total CTCs (≥3): 62%/89.5%, 0.77 Total CTCs or AFP: AUC = 0.82
**Prognosis**				
**Study**	**CTCs Definition**	**HCC Patients**	**Stage/Treatment**	**Main Findings**
Vona et al., 2004 [203]	Size (diameter > 25 μm)	*N* = 44	Stage: 39% multinodular, 39% tumor ≤3 cm, 45% PVT, no EHS Treatment: NR	Patients with CTCs/circulating tumor microemboli had poorer OS (*p* = 0.01) No significant association with survival in multivariate analysis.
Fan et al., 2011 [208]	CD45 (−) CD90 (+) CD44 (+)	*N* = 82	TNM stage I/II/III/IV: 5%/34%/34%/27% Treatment: LR	CTCs predicted recurrence (sensitivity/specificity): 65.9%/80.5% CTCs (>0.01%) independently associated with poorer: Median recurrence-free survival (6.0 vs. >46.5 months) 2-years recurrence-free survival (22.7% vs. 64.2%) 2-year OS (58.5% vs. 94.1%) (*p* < 0.001 for all)
Liu et al., 2013 [210]	CD45 (−) ICAM-1 (+)	*N* = 60	Stage: tumor size >5 cm 72%, multifocal 12% Treatment: LR	High proportion of ICAM-1 (+) CTCs associated with: Poorer DFS: adjusted HR = 7.15 (2.99–17.05) No independent association with OS: adjusted HR = 2.28 (0.95–7.82)
Nel et al., 2013 [237]	CTCs: CD45 (−), DAPI (+), EpCAM (+), ASGPR1 (+) Mesenchymal: either N-cadherin (+) or vimentin (+) Epithelial: pan-CK (+) Mixed: CK (+) and either N-cadherin (+) or vimentin (+)	*N* = 11	Stage: NR Treatment: various (SIRT in 45%)	Vimentin (+)/CK (+) ratio >0.5 associated with a longer TTP: 1 vs 15 months (*p* = 0.03) N-cadherin (+)/CK (+) ratio <0.1 associated with a shorter TTP: 1 vs 15 months (*p* = 0.03)
Sun et al., 2013 [204]	CellSearch™	*N* = 123	BCLC stage 0-A/B-C: 82%/18% Treatment: LR	Presence of CTCs (>2/7.5 mL) before surgery associated with: Increased risk of recurrence: adjusted HR = 5.20 (2.65–10.21)
Cheng et al., 2013 [209]	Magnetic cell sorting and PCR for Lin28B	*N* = 96	BCLC stage A/B-C: 55%/45% Treatment: LR	Lin28B positive CTCs associated with: Decreased RFS: adjusted HR = 2.25 (1.01–4.99) Early recurrence (<1 year): adjusted HR = 2.65 (1.02–6.86); also true in earlier stages
Schulze et al., 2013 [205]	CellSearch™	*N* = 59	BCLC stage A/B/C: 15%/53%/32% Treatment: NR	Detection of CTCs was associated with lower OS at the Kaplan-Meier analysis (*p* = 0.02)
Guo et al., 2014 [202]	CellSearch™ and quantitative PCR for EpCAM in CD45 (-) cells	*N* = 299	Stage: NR Treatment: LR 53%, TACE 25%, RT 22%	EpCAM mRNA (+) CTCs associated with worse outcomes Surgery: shorter TTR; adjusted HR = 2.9 (1.6–5.3) TACE: shorter PFS; unadjusted HR = 3.8 (1.4–10) RT: shorter PFS; unadjusted HR = 5.1 (1.4–18.5)
Nel et al., 2014 [238]	CD45 (−), EpCAM (+), DAPI (+), pan-CK (+) and IGFBP1 mRNA (+)	*N* = 25	TNM stage II/III/IV: 28%/48%/24% Treatment: SIRT	Low expression of IGFBP1 mRNA in CTCs discriminate progression from disease control (sensitivity 80%, specificity 80%, AUC = 0.8). Low IGFBP1 mRNA in CTCs correlated with shorter TTP (*p* = 0.04)
Li et al., 2016 [222]	Density-based, CD45 (−), pan-CK (+) and either pAkt1/2/3 or pERK1/2 (+)	*N* = 109	Stage: advanced Treatment: sorafenib	High proportion of pERK (+) pAkt (−) CTCs associated with longer PFS: adjusted HR = 9.39 (3.24–27.19)
Ogle et al., 2016 [195]	CD45 (−), morphology, size	*N* = 69	BCLC stage A/B/C/D: 16%/7%/73%/4% Treatment: LT 6%, LR 4%, ABL 10%, IAT 39%, sorafenib 13%, BSC 28%	Presence of CTCs (>1/4 mL) at any time (*N* = 69): Shorter OS: adjusted HR = 2.34 (1.015.43) Shorter TTP (*p* = 0.006) Presence of CTCs post-treatment (*N* = 29): Shorter OS: adjusted HR = 6.16 (1.71–22.33) Shorter TTP (*p* = 0.002)
Zhou et al., 2016 [232]	EpCAM mRNA (+)	*N* = 49	BCLC stage 0-A/B-C: 90%/10% Treatment: LR	High EpCAM mRNA (+) CTCs associated with increased risk of recurrence: adjusted HR = 6.69 (1.94–22.88)
von Felden et al., 2017 [206]	CellSearch™	*N* = 57	BCLC stage A/B: 92%/8% Treatment: LR	CTCs status was independently associated with increased risk of recurrence: adjusted HR = 3.1 (1.0–9.4)
Guo et al., 2018 [200]	CTC detection panel: PCR for EpCAM, CD133, CD90 and CK19	*N* = 395	Training: BCLC stage 0-A: 66% Treatment: LR 98%, TACE 2% Validation: BCLC stage 0-A: 48% Treatment: LR 67%, TACE 33%	CTC detection panel was associated with shorter TTR: Training cohort: adjusted HR = 2.69 (1.62–4.48) Validation cohort: adjusted HR = 3.13 (1.36–7.19) Association remained significant in patients with negative AFP and with early-stage (BCLC 0-A) tumor
Qi et al., 2018 [211]	Can Patrol™	*N* = 112	BCLC stage 0/A/B/C: 10%/39%/21%/30% Treatment: LR	CTCs associated with HCC recurrence: CTC count: adjusted HR = 1.02 (1.01–1.04) Mesenchymal CTC percentage: adjusted HR = 1.02 (1.01–1.03) Mesenchymal > epithelial CTC percentage: adjusted HR = 1.00 (0.99–1.02) Mesenchymal = epithelial CTC percentage, mesenchymal < epithelial CTC percentage, epithelial CTC percentage not associated with recurrence at univariate analysis.
Sun et al., 2018 [226]	CellSearch™	*N* = 73	BCLC stage 0-A/B-C: 77%/23% Treatment: LR	Presence of CTCs in different vascular sites. Association with intrahepatic recurrence: Peripheral veins: adjusted HR = 0.77 (0.14–5.19) Peripheral arteries: adjusted HR = 2.54 (0.87–7.42) Peripheral veins CTCs with clusters: adjusted HR = 3.48 (1.40–8.61) Association with lung metastasis: Hepatic vein CTCs: adjusted HR = 0.59 (0.04–9.54) Intrahepatic inferior vena cava CTCs: adjusted HR = 0.67 (0.10–4.40) Hepatic vein CTCs with clusters: adjusted HR = 42.2 (3.73–477.8)
Wang et al., 2018 [239]	CanPatrol™	*N* = 62	BCLC stage 0-A/B-C: 37%/63% Treatment: LR	Association with early recurrence: Total CTCs: unadjusted HR = 2.95 (1.18–7.35); NS after adjustment Mesenchymal CTCs: unadjusted HR = 4.74 (2.04–11.01); adjusted HR = 3.45 (1.39–8.56) Mixed CTCs: unadjusted HR = 2.94 (1.31–6.59); NS after adjustment
Yu et al., 2018 [215]	CellSearch™	*N* = 139	BCLC stage 0+A/B+C: 40%/60% Treatment: LR	4 categories: 1) persistently (+); 2) preoperatively (+) but postoperatively (−); 3) preoperatively (−) but postoperatively (+); 4) persistently (−). For a 1-point increase in category: DFS: adjusted HR = 0.53 (0.41–0.68) OS: adjusted HR = 0.48 (0.36–0.66)
Ye et al., 2018 [240]	CanPatrol™	*N* = 42	BCLC stage A-B/C-D: 81%/19% Treatment: LR	Pre-operative CTC count not associated with OS and PFS Post-operative CTC count (>5): Poorer PFS: adjusted HR = 6.89 (1.64–29.0) No independent association with OS: adjusted HR = 15.65 (0.80–304.64) Increase of post-operative CTC count: Poorer PFS: adjusted HR = 39.58 (4.22–371.64)
Wang et al., 2018 [213]	SE-iFISH	*N* = 14	Stage: NR Treatment: NR	Detection of small CTCs with triploid chromosome 8 showed shorter DFS (*p* = 0.007); HR not reported
Court et al., 2018 [241]	NanoVelcro™	N = 80	BCLC stage A/B/C/D: 18%/28%/43%/11% Treatment: ABL, TACE, SIRT, systemic therapy, BSC	Total CTCs were associated with: Shorter TTR in patients with early stage: univariate HR = 9.7 (2.08–45.19); no significant association in multivariate. Shorter PFS in patients with advanced disease: univariate HR = 2.09 (1.11–3.96); multivariate HR =2.09 (1.11–3.96) Vimentin (+) CTCs independently associated with: Poorer OS: adjusted HR = 2.21 (1.38–3.56) Poorer PFS in patients with advanced disease: adjusted HR = 2.16 (1.33–4.42) Trend toward fast TTR in patients with early stage: adjusted HR = 2.45 (0.91–6.57)
Shen et al., 2018 [242]	CellSearch™	*N* = 97	BCLC stage A-B/C: 56%/44% Treatment: TACE	CTC count independently predicted OS: High vs. low level group: adjusted HR = 2.82 (1.22–6.53) Intermediate vs. low group: adjusted HR = 1.30 (0.63–2.69)
Ha et al., 2019 [214]	Tapered slit platform (detection based on size and morphology)	*N* = 105	BCLC stage 0/A: 19%/81% Treatment: LR	Presence of pre- and post-operative CTCs not associated with recurrence. Positive ΔCTC (increase of CTC after surgery): Shorter RFS: adjusted HR = 2.28 (1.06–4.90) No associations with OS
Hamaoka et al., 2019 [243]	Glypican-3 (+)	*N* = 85	Stage: median tumor number 1 and median size 25 mm Treatment: LR	CTCs associated with: Higher risk of microscopic portal vein invasion: adjusted OR = 14.6 (3.3–106.0) Shorter DFS (*p* = 0.02) Shorter OS (*p* = 0.047)
Wu et al., 2019 [244]	CD45 (−) and abnormal chromosome 8 amplification by FISH	*N* = 155	BCLC stage A/B/C: 38%/14%/48% Treatment: TACE	Presence of pre-TACE CTCs associated with poorer OS: adjusted HR = 2.84 (1.41–5.73)
Chen et al., 2020 [218]	CD45 (-) and imFISH	*N* = 50	TNM stage I/II/III/IV: 8%/32%/58%2% Treatment: LT	CTCs detection was associated with recurrence post-LT: adjusted HR = 5.41 (1.13–25.87)
Zhou et al., 2020 [245]	Size and deformability	*N* = 137	BCLC stage 0-A/B-C: 57%/43% Treatment: LR	Presence of CTCs: Independently associated with microvascular invasion: adjusted HR = 1.76 (1.34–2.30) Shorter OS (19.2 months vs. not reached; *p* = 0.005)
Winograd et al., 2020 [223]	CD45 (−), DAPI (+), CK (+), PD-L1 (+)	*N* = 87	BCLC stage A/B/C/D: 25%/25%/41%/8% Treatment: various; checkpoint inhibitors 14.3%	Detection of CTCs expressing PD-L1:Associated with poorer OS (≥4 PD-L1 (+) CTCs): adjusted HR = 3.22 (1.33–7.79) Predicted response to checkpoint inhibitors
Wang et al., 2020 [246]	CellSearch™	*N* = 344	BCLC stage 0-A/B-C: 73.8%/26.2% Treatment: LR ± adjuvant TACE	After propensity score matching, in CTC positive patients’ adjuvant TACE provide benefits in: TTR (45.8 vs. 9.8 months, *p* < 0.001) OS (not reached vs. 36.4 months; *p* < 0.001)
Wang et al., 2020 [219]	ChimeraX^®^-i120 platform	*N* = 193	Stage: Milan-in 60% Treatment: LT	Post-operative CTC count ≥1 independently associated with tumor recurrence: adjusted HR = 2.67 (1.50–4.74)

^†^ Cohort of Guo et al., 2014 [202] and Guo et al., 2018 [200] may overlap. Abbreviations: AFP, alpha-fetoprotein; ABL, ablation; AUC, area under the curve; BCLC, Barcelona Clinic Liver Cancer; BSC, best supportive care; DC, disease control; DFS, disease-free survival; EHS, extrahepatic spread; HBV, hepatitis B virus; HCC, hepatocellular carcinoma; HCV, hepatitis C virus; HR, hazard ratio; IAT, intra-arterial therapies; LR, liver resection; LT, liver transplantation; OS, overall survival; OR, odds ratio; NS, not significant; NR, not reported; PFS, progression-free survival; PVT, portal vein thrombosis; RFS, recurrence-free survival; RT, radiotherapy; SIRT, selective internal radiation therapy; TACE, transarterial chemoembolization; TTP, time to progression; TTR, time to recurrence.

## 5. Conclusions and Future Perspectives

The identification of reliable non-invasive biomarkers that could allow a personalized management of HCC patients has become a key priority in the last years. Circulating markers that can integrate or eventually replace percutaneous liver biopsy, overcoming its limitations, are crucial. In addition, HCC detection at early stages, when it is susceptible to potentially curative treatments, and prediction of response to therapy are critical to improve patient survival. Although fewer data are available for HCC compared to other malignancies, numerous recent publications demonstrated very interesting and promising results regarding liquid biopsy role in diagnosis, prognosis, and prediction of response to treatment. cfDNA, cfRNA, EVs, and CTCs emerged as attractive liquid biopsy candidates because they fulfil many of the major characteristics of an ideal biomarker. To date, the closest approach to reaching the introduction in clinical practice, after the necessary large and prospective studies, is cfDNA methylation profiling for early detection of HCC in patients at risk. Mutational profiling of cfDNA and CTCs analyses are dependent on tumor burden and therefore likely more useful in intermediate or advanced settings as prognostic and predictive tools. Even tough fewer data are currently available, the analysis of EVs could provide biomarkers at every HCC stage and has the advantage to provide functional information (e.g., interactions between cancer cells and tumor microenvironment or distant cells).

Although the large amount of encouraging data collected in recent years predict a bright future for liquid biopsy in HCC, its widespread clinical application is yet not on the horizon. The majority of data supporting its utility derives from proof-of-concept studies, mainly retrospective, and not validated by different researchers. The main limitation that hinders the routine application of liquid biopsy is the lack of standardization, absence of accepted standard operating procedures, and the lack of comparability between existing approaches [47]. The standardization of pre-analytical, analytical, and post-analytical variables should be addressed. Considering for instance cfDNA analysis, the avoidance of white blood cells (WBC) lysis during blood collection and processing is important to prevent dilution of tumor circulating fragments with non-tumoral DNA (pre-analytical phase). Moreover, transportation, processing and storage temperature are also critical, impacting on WBC stability and cfDNA degradation. Since cfDNA has a short half-life and there are time-dependent changes of DNA in blood collection tubes (because of the degradation from DNase activity), plasma should be isolated within an hour from collection (analytical phase). Considered the relevance of these and other variables on the final results, the standardization of methodological protocols is an essential step to take in order to integrate liquid biopsy in the everyday clinical practice.

With the aim of identifying clinically useful diagnostic biomarkers, studies should include as controls only patients at risk of developing HCC (i.e., cirrhotics or high risk chronic hepatitis patients), who represent the ideal target for surveillance [5]. This is not trivial, also considering that it could make more difficult the identification of specific diagnostic biomarkers. In fact, chronic hepatitis and cirrhosis are pre-cancerous conditions in which some of the molecular modifications found in overt HCC are already in place. For instance, during the progression of liver damage the pattern of DNA methylation changes over time in multiple hepatic cell types, and the release of methylated cfDNA from dying hepatocytes has been demonstrated to be a useful approach to evaluate fibrosis grade [247,248]. In order to have a chance of being introduced in clinical practice, liquid biopsy biomarkers should be specific enough to distinguish early-stage HCC from simple cirrhosis, a condition in which the molecular pathways leading to cancer may be already at least in part activated. In addition, when tumor burden is low, highly sensitive tests are necessary to overcome the limitation posed by the small amount of circulating cancer by-products. Even though these new liquid biopsy strategies represent very promising tools, another not negligible consideration should be done about their costs. While currently used biomarkers (AFP) are measured with unexpensive and simple methods, EVs isolation and analysis, cfDNA mutational profiling and epigenetic analysis, and CTCs enrichment methods require devoted personnel and are all costly and time consuming. Nevertheless, such limitations will likely be overcome by advances in technology that will make these determinations easier and accessible to most laboratories.

Once these new generation reliable biomarkers will be developed and validated, the final step will be to determine the optimal way to integrate them in the clinical management of patients with HCC. The replacement of currently used tools in the management of HCC patients by liquid biopsy biomarkers is unrealistic, but they will likely be integrated in the process, providing a stronger predictive power. An interesting approach in surveillance, which remains to be evaluated in ad hoc studies, could be the combined evaluation of liquid biopsy biomarkers with the currently used periodic liver ultrasonography. Given the possibility of minimally invasive repeated sampling, liquid biopsies can enable real-time monitoring of disease during therapy and could supplement imaging information to provide a more careful assessment of the tumor. Hopefully, in the future, the analysis of circulating HCC by-products will also allow personalized molecular targeted therapy. In order to achieve these important goals, not only prospective observational trials should be conducted, to correlate liquid biopsy biomarkers with clinical outcome, but also interventional studies, in which cfDNA, EVs, and CTCs analysis will prompt therapeutic decisions, are necessary.

## Supplementary Materials

The following are available online at https://www.mdpi.com/article/10.3390/cancers13092274/s1, Table S1: Studies on use of microRNAs (miRNAs) as biomarkers in HCC.
