# Supplementary material for: Liquid Biopsy in Hepatocellular Carcinoma: Where Are We Now?
Source: Cancers (Basel). 2021 May 10;13(9):2274. doi: 10.3390/cancers13092274 (PMC8126224; doi:10.3390/cancers13092274)
Supplement: Supplementary file 1 [file cancers-13-02274-s001.zip › cancers-1206288-supplementary.pdf]

cancers

MDPI

Review

Liquid Biopsy in Hepatocellular Carcinoma: Where Are We Now?

Filippo Pelizzaro [1], Romilda Cardin [1], Barbara Penzo [1], Elisa Pinto [1], Alessandro Vitale [2], Umberto Cillo [2], Francesco Paolo Russo [1] and Fabio Farinati [1,*]

1 Gastroenterology Unit, Department of Surgery, Oncology and Gastroenterology, University of Padua, 35128 Padua, Italy; filippo.pelizzaro@unipd.it (F.P.); romilda.cardin@unipd.it (R.C.); barbara.penzo@aopd.veneto.it (B.P.); elisa.pinto@aopd.veneto.it (E.P.); francescopaolo.russo@unipd.it (F.P.R.)
2 Hepatobiliary Surgery and Liver Transplantation Unit, Department of Surgery, Oncology and Gastroenterology, University of Padua, 35128 Padua, Italy; alessandro.vitale@unipd.it (A.V.); cillo@unipd.it (U.C.)
* Correspondence: fabio.farinati@unipd.it

Citation: Pelizzaro, F.; Cardin, R.; Penzo, B.; Pinto, E.; Vitale, A.; Cillo, U.; Russo, F.P.; Farinati, F. Liquid Biopsy in Hepatocellular Carcinoma: Where Are We Now? *Cancers* **2021**, *13*, 2274. https://doi.org/10.3390/cancers13092274

Academic Editor: Anna Kakehashi

Received: 16 April 2021
Accepted: 6 May 2021
Published: 10 May 2021

Publisher's Note: MDPI stays neutral with regard to jurisdictional claims in published maps and institutional affiliations.

Copyright: © 2021 by the authors. Licensee MDPI, Basel, Switzerland. This article is an open access article distributed under the terms and conditions of the Creative Commons Attribution (CC BY) license (http://creativecommons.org/licenses/by/4.0/).

Supplementary Table 1. Studies on use of microRNAs (miRNAs) as biomarkers in HCC.

Diagnosis				
**Study**	**Type of miRNA**	**Number of patients**	**Comparator**	**Main findings (sensitivity/specificity, AUC)**
Zhou et al, 2011 [1]	miRNA panel: miR-122, miR-192, miR-21, miR-223, miR-26a, miR-27a and miR-801	Training phase: 204 HCC, 60 LC, 75 CLD (HBV), healthy subjects 68<br>Validation phase: 196 HCC, 56 LC, 72 CLD (HBV), 66 healthy subjects	AFP (cut-off 400 ng/mL)	Training cohort: 68.6%/90.1%, 0.864<br>Validation cohort: 81.8%/83.5%, 0.888<br>AUC according to BCLC stage: 0 = 0.888, A = 0.888, B = 0.901, and C = 0.881<br>In AFP <400 ng/mL: 77.7%/84.5%, 0.879<br>In AFP ≥400 ng/mL: 87.7%/83.5%, 0.910<br>HCC vs. healthy subjects: 83.2%/93.9%, 0.941<br>HCC vs. CLD: 79.1%/76.4%, 0.842<br>HCC vs. LC: 75%/91.1%, 0.884
Tomimaru et al, 2012 [2]	miR-21	126 HCC<br>30 CLD<br>50 healthy subjects	AFP (cut-off 19 ng/mL in HCC vs. CLD and 6 ng/mL in HCC vs. healthy subjects)	HCC vs. CLD:<br>miR-21: 61.1%/83.3%, 0.773<br>AFP: 59.5%/83.3%, 0.743<br>miR-21 + AFP: 81%/76.7%, 0.823<br>HCC vs. healthy subjects:<br>miR-21: 87.3%/92%, 0.953<br>AFP: 77.8%/96%, 0.882<br>miR-21 + AFP: 92.9%/90%, 0.971
Lin et al, 2015 [3]	miRNA classifier: miR-29a, miR-29c, miR-133a, miR-143, miR-145, miR-192, and miR-505	Training cohort: 108 HCC, 47 LC, 51 CLD (HBV), 51 healthy subjects<br>Validation cohort: 2020 HCC, 181 CLD + LC, 108 healthy subjects	AFP (cut-off 20 ng/mL)	Validation cohort 1:<br>miRNA classifier: 74.5%/88.9%, 0.817<br>AFP: 56.9%/84.9%, 0.709 (p<0.05 vs. miRNA classifier)<br>Validation cohort 2:<br>miRNA classifier: 85.7%/91.1%, 0.884<br>AFP: 59.2%/100%, 0.796 (p<0.05 vs. miRNA classifier)<br>miRNA classifier vs. AFP (AUC):<br>Small HCC: 0.833 vs. 0.727 (p<0.05)<br>Early-stage HCC: 0.824 vs. 0.754 (p<0.05)<br>miRNA classifier in AFP negative patients (AUC): 0.825
El-Tawdi et al, 2016 [4]	lncRNA-CTBP +miR-16-2 + miR-21-5p + LAMP2	78 HCC<br>36 CLD (HCV)<br>44 healthy subjects	NR	HCC vs. CLD + healthy subjects: 79.5%/100%, 0.938
Amr et al, 2016 [5]	miR-21 and miR-199a	23 HCC<br>17 CLD	AFP	miR-21: 100%/81.2%, 0.943<br>miR-199a: 54.5%/100%, 0.856<br>AFP: 100%/69.2%, 0.832
Okajima et al, 2016 [6]	miR-224	87 HCC<br>55 healthy subjects	AFP (cut-off 20 ng/mL)<br>DCP (cut-off 40 mAU/mL)	93.1%/80.0%, 0.908<br>Early-stage HCC (TNM stage I) vs. healthy subjects: AUC=0.0899<br>miR-224 in the detection of tumor <18 mm (sensitivity, AUC):<br>First cohort: 80%, 0.802 (DCP 45%, 0.741; AFP 50%, 0.475)<br>Second cohort: 63.6%, 0.731 (DCP 50%, 0.595; AFP 20%, 0.726)

Zhuang et al, 2016 [7]	miR-21, miR-26a and miR-101	52 HCC<br>42 CLD<br>43 healthy subjects	AFP (cut-off NR)	HCC vs. healthy subjects:<br>miR-21 + miR-26a + miR-101: 88.2%/58.5%, 0.803<br>miR-21 + miR-26a + miR-101 + AFP: 87%/78%, 0.914<br>HCC vs. CLD:<br>miR-26a + miR-101: 70.6%/80.2%, 0.822<br>miR-26a + miR-101 + AFP: 72.5%/86.7%, 0.854
Zekri et al, 2016 [8]	Several miRNAs	192 HCC<br>96 LC<br>96 CLD (HCV)<br>95 healthy subjects	AFP (cut-off NR)	HCC vs. healthy subjects:<br>miR-122 + miR-885-5p + miR-29b + AFP: AUC = 1<br>HCC vs. LC:<br>miR-122 + miR-885-5p + miR-221 + miR-22 + AFP: AUC = 0.982<br>HCC vs. CLD:<br>miR-22 + miR-199a-3p + AFP: AUC = 0.988
Shi et al, 2017 [9]	miR-106b	25 HCC<br>310 non-HCC	NR	Patients with HCC had higher serum miR-106b levels.<br>90%/66.7% (0.855)
Guo et al, 2017 [10]	miR-21	175 HCC<br>64 CLD (HBV)<br>78 LC<br>136 healthy subjects	AFP (cut-off 16.42 ng/mL)	HCC vs. LC:<br>miR-21: 80.8%/72.9%, 0.814<br>AFP: 70.4%/71.5%, 0.686<br>HCC vs. CLD:<br>miR-21: 76.9%/85.7%, 0.789<br>AFP: 59.3%/69.7%, 0.634
Zhang et al, 2017 [11]	miRNA panel: miR-92-3p, miR-107 and miR-3126-5p	115 HCC<br>40 healthy subjects	AFP (cut-off NR)	miR-3126-5p: AUC = 0.881<br>miR-107: AUC = 0.730<br>miR-92a-3p: AUC = 0.705<br>miRNA panel: AUC = 0.969 vs. AFP: AUC=0.848<br>miRNA panel + AFP: AUC = 0.994
Moshiri et al, 2018 [12]	miR-106b-3p, miR-101-3p and miR-1246	62 HCC<br>41 LC<br>25 healthy subjects	NR	HCC vs. healthy subjects: 100%/100%, 1.00<br>HCC vs. LC: 100%/92.9%, 0.99s
An et al, 2018 [13]	miR-375, miR-10a, miR-122 and miR-423	84 HCC<br>84 normal controls	NR	miR-375: AUC = 0.918<br>miR-10a: AUC =0.838<br>miR-122: AUC = 0.871<br>miR-423: AUC =0.898<br>miR-375, miR-10a, miR-122 and miR-423: AUC = 0.995
Han et al, 2019 [14]	miR-148a	155 HCC<br>96 LC<br>95 healthy subjects	AFP (cut-off NR)	HCC vs. healthy subjects: 97.9%/92.9%, 0.980<br>HCC vs. LC:<br>AFP: 88.4%/84.4%, 0.941<br>miR-148a: 89.6%/89%, 0.919 (in patients with low AFP: 90.6%/92.6%, 0.949)
Weis et al, 2019 [15]	miR-122-5p, miR-486-5p and miR-142-3p	20 HCC<br>20 LC<br>20 CLD	AFP (cut-off 20 ng/mL)	miR-122 + miR-486 + miR-142: 80%/95%, 0.94<br>AFP: 25%/90%, 0.64 (p=0.06 vs. miRNA panel)<br>miR-122 + miR-486 + miR-142 + AFP: 0.94

Yamamoto et al, 2020 [16]	8 miRNA panel: miR-320b, miR-663a, miR-4448, miR-4651, miR-4749-5p, miR-6724-5p, miR-6877-5p, and miR-6885-5p	353 HCC<br>93 LC<br>46 CLD<br>1033 healthy subjects	AFP (cut-off 10 ng/mL)<br>DCP (cut-off 40 mAU/mL)	HCC vs. healthy subjects: 97.7%/98.4%, 1.00<br>HCC vs. CLD + LC: 97.7%/94.7%, 0.99<br>In TNM stage I, the 8 miRNAs panel: 100%/94.7%, 1.00
**Prognosis**				
**Study**	**Type of miRNA**	**HCC patients**	**Stage /Treatment**	**Main findings**
Koberle et al, 2013 [17]	miR-1 and miR-122	N = 195	BCLC stage A/B/C/D: 24%/39%/30%/7%<br>Treatment: LR 9%, ABL+IAT 53%, SOR 24%, LT 11%	At univariate analysis, longer survival in:<br>High miR-1 group: HR = 0.44 (0.23-0.83)<br>High miR-122 group: HR = 0.49 (0.25-0.96)<br>High miR-1 was an independent predictor of better OS: adjusted HR = 0.45 (0.24-0.86)
Xu et al, 2015 [18]	miR-122	N = 122	Stage: NR<br>Treatment: NR	High miR-122 associated with longer OS: adjusted HR = 0.26 (0.14-0.47)
Cho et al, 2015 [19]	miR-122	N = 120	TNM stage I+II/III+IV: 73.3%/26.7%<br>Treatment: LR 52.5%, ABL 47.5%	miR-122 levels were not associated with OS in the entire cohort and in LR patients.<br>In ABL patients, high miR-122 associated with shorter OS: adjusted HR = 2.67 (1.12-6.35).
Okajima et al, 2016 [6]	miR-224	N = 87	TNM stage I/II-IV: 71%/29%<br>Treatment: LR	miR-224 was significantly reduced in post-LR samples (p=0.006).<br>Tumor >2 cm, advanced stage and presence of recurrences correlated with high levels of miR-224 (p=0.0005, 0.04 and 0.003, respectively).
Cho et al, 2017 [20]	miR-21, miR-26a and miR-29a	N = 120	TNM stage I+II/III+IV: 73.3%/26.7%<br>Treatment: LR 52.5%, ABL 47.5%	Poorer DFS was demonstrated for:<br>Low miR-26a levels: adjusted HR = 1.72 (1.04-2.83)<br>Low miR-29a levels: adjusted HR = 1.75 (1.04-2.94)<br>Shorter LT-free survival was demonstrated for:<br>Low miR-26a: adjusted HR = 3.41 (1.32-8.82)<br>Low miR-29a: adjusted HR = 2.75 (1.10-6.85)
Fornari et al, 2017 [21]	miR-221	N = 90 (50 in training set and 43 in validation set)	Stage: advanced HCC without extrahepatic metastases<br>Treatment: sorafenib	Both in training and in validation cohorts:<br>Higher pre-treatment miR-221 levels in non-responders vs. responders.<br>After 2 months of treatment: increase of miR-221 in responders; non-significant decrease in non-responders.
Nishida et al, 2017 [22]	miR-181a-5p and miR-339-5p	N = 53	BCLC stage A/B/C: 15%/28.3%/56.6%<br>Treatment: sorafenib	miR-181a-5p independently predicted DC: adjusted HR = 0.14 (0.01–0.66).<br>High miR-181a-5p was associated with longer OS: adjusted HR = 0.27 (0.07-0.82).
Kim et al, 2018 [23]	miR-21, miR-26a and miR-29a-3p	N = 198	BCLC stage: A-B/C-D: 73.2%/26.8%<br>Treatment: TACE	High miR-21, high miR-26a and low miR-29a-3p levels associated with overall TACE refractoriness (no after adjustment for confounders).<br>miRNA combination panel (high miR-21 and miR-26a and low miR-29a-3p) independently predict early TACE refractoriness: adjusted HR = 2.32 (1.08-4.99).

Chuma et a, 2019 [24]	miR-1246	N = 121	BCLC stage 0/A/B: 27.3%/67.8%/5%<br>Treatment: LR	miR-1246 levels higher in patients with early tumor recurrence.<br>High levels of miR1246 associated with:<br>Early tumor recurrence: adjusted HR = 3.42 (1.33-8.82)<br>Shorter DFS (p<0.001)<br>Shorter OS (p<0.001); adjusted HR = 2.78 (1.53-5.07)
Ali et al, 2019 [25]	miR-133b, miR-26a, miR-107 and miR-106	N = 51	Stage: single tumor 57%<br>Treatment: TACE	Baseline miR-106b, miR-107 and miR-133b elevated in TACE-responders; miR-26a in non-responders (all p<0.001).<br>Prediction of TACE response (sensitivity/specificity, AUC):<br>miR-26a: 100%/100%, 1.00 in CR vs. NR; 94%/83%, 0.958 in CR vs. PR<br>miR-133b: 100%/94%, 0.997 in CR vs. NR; 93%/88%, 0.919 in CR vs. PR; 94%/83%, 0.935 in PR vs. NR<br>miR-26a + miR-133 (AUC): 1.00 in CR vs. NR; 0.997 in PR vs. NR; 0.919 in CR vs. PR; 0.998 in CR+PR vs. NR
Ning et al, 2019 [26]	miR-155, miR-96 and miR-99a	N = 30	TNM stage I+II/III+IV: 40%/60%<br>Treatment: LR	Decreased OS in patients with:<br>High miR-155 levels (p=0.004)<br>High miR-96 levels (p=0.02)<br>miR-99a levels were not significantly associated with OS.
Jin et al, 2019 [27]	miR-128, miR-139-5p, miR-382-5p, miR-410, miR-424-5p and miR-101-3p	N = 116	Stage: multifocal 29%<br>Treatment: NR	Higher expression of miR-128, miR-139-5p, miR-382-5p and miR-410 and lower levels of miR-424-5p and miR-101-3p associated with worse prognosis.
Han et al, 2019 [14]	miR-148a	N = 155	Stage: metastases 30%<br>Treatment: TACE or ABL	miR-148a increased significantly after treatment (p<0.0001).
Teufel et al, 2019 [28]	miR-30a, miR-122, miR-125b, miR-200a, miR-374b, miR-15b, miR-107, miR-320 and miR-645	N = 243	BCLC stage A/B/C: <1%/14%/86% (in RESORCE trial)<br>Treatment: regorafenib	Increased miR-30a, miR-122, miR-125b, miR-200a, and MIR374B, decreased miR-15b, miR-107, and miR-320b, and absence of miR-645 predictive of survival benefit.<br>miR-15b, miR-320b, and miR-200a were prognostic for OS (p<0.05)
Loosen et al, 2020 [29]	miR-193a-5p	N = 41	TNM stage T1-2/T3-4: 75%/25%<br>Treatment: LR 19.5%, LT 80.5%	High levels of miR-193a-5p independently associated with poorer OS: adjusted HR = 3.71 (1.35-10.16)
Pratedrat et al, 2020 [30]	miR-223-3p	N = 70	BCLC stage 0-A/B/C-D: 30%/34.3%/35.7%<br>Treatment: NR	High levels of miR-223-3p independently associated with poorer OS: adjusted HR = 6.61 (2.36-18.55)

Abbreviations: ABL, ablation; AFP, alpha-fetoprotein; AUC, area under the curve; BCLC, Barcelona Clinic Liver Cancer; CLD, chronic liver disease; CR, complete response; DC, disease control; DCP, des-γ-carboxyprothrombin; DFS, disease-free survival; HCC, hepatocellular carcinoma; HR, hazard ratio; IAT, intrarterial therapies; LC, liver cirrhosis; LR, liver resection; LT, liver transplantation; miRNA, microRNA; NR, not reported; OS, overall survival; PR, partial response; SOR, sorafenib; TACE, trans-arterial chemoembolization.

References

1. Zhou, J.; Yu, L.; Gao, X.; Hu, J.; Wang, J.; Dai, Z.; Wang, J.-F.; Zhang, Z.; Lu, S.; Huang, X.; et al. Plasma microRNA panel to diagnose hepatitis B virus-related hepatocellular carcinoma. *J. Clin. Oncol.* **2011**, *29*, 4781–4788, doi:10.1200/JCO.2011.38.2697.
2. Tomimaru, Y.; Eguchi, H.; Nagano, H.; Wada, H.; Kobayashi, S.; Marubashi, S.; Tanemura, M.; Tomokuni, A.; Takemasa, I.; Umeshita, K.; et al. Circulating microRNA-21 as a novel biomarker for hepatocellular carcinoma. *J. Hepatol.* **2012**, *56*, 167–175, doi:10.1016/j.jhep.2011.04.026.
3. Lin, X.J.; Chong, Y.; Guo, Z.W.; Xie, C.; Yang, X.J.; Zhang, Q.; Li, S.P.; Xiong, Y.; Yuan, Y.; Min, J.; et al. A serum microRNA classifier for early detection of hepatocellular carcinoma: A multicentre, retrospective, longitudinal biomarker identification study with a nested case-control study. *Lancet Oncol.* **2015**, *16*, 804–815, doi:10.1016/S1470-2045(15)00048-0.
4. El-Tawdi, A.H.F.; Matboli, M.; Shehata, H.H.; Tash, F.; El-Khazragy, N.; Azazy, A.E.S.M.; Abdel-Rahman, O. Evaluation of Circulatory RNA-Based Biomarker Panel in Hepatocellular Carcinoma. *Mol. Diagnosis Ther.* **2016**, *20*, 265–277, doi:10.1007/s40291-016-0200-9.
5. Amr, K.S.; Ezzat, W.M.; Elhosary, Y.A.; Hegazy, A.E.; Fahim, H.H.; Kamel, R.R. The potential role of miRNAs 21 and 199-a in early diagnosis of hepatocellular carcinoma. *Gene* **2016**, *575*, 66–70, doi:10.1016/j.gene.2015.08.038.
6. Okajima, W.; Komatsu, S.; Ichikawa, D.; Miyamae, M.; Kawaguchi, T.; Hirajima, S.; Ohashi, T.; Imamura, T.; Kiuchi, J.; Arita, T.; et al. Circulating microRNA profiles in plasma: Identification of miR-224 as a novel diagnostic biomarker in hepatocellular carcinoma independent of hepatic function. *Oncotarget* **2016**, *7*, 53820–53836, doi:10.18632/oncotarget.10781.
7. Zhuang, C.; Jiang, W.; Huang, D.; Xu, L.; Yang, Q.; Zheng, L.; Wang, X.; Hu, L. Serum miR-21, miR-26a and miR-101 as potential biomarkers of hepatocellular carcinoma. *Clin. Res. Hepatol. Gastroenterol.* **2016**, *40*, 386–396, doi:10.1016/j.clinre.2015.11.002.
8. Zekri, A.R.N.; Youssef, A.S.E.D.; El-Desouky, E.D.; Ahmed, O.S.; Lotfy, M.M.; Nassar, A.A.M.; Bahnassey, A.A. Serum microRNA panels as potential biomarkers for early detection of hepatocellular carcinoma on top of HCV infection. *Tumor Biol.* **2016**, *37*, 12273–12286, doi:10.1007/s13277-016-5097-8.
9. Shi, B.M.; Lu, W.; Ji, K.; Wang, Y.F.; Xiao, S.; Wang, X.Y. Study on the value of serum miR-106b for the early diagnosis of hepatocellular carcinoma. *World J. Gastroenterol.* **2017**, *23*, 3713–3720, doi:10.3748/wjg.v23.i20.3713.
10. Guo, X.; Lv, X.; Lv, X.; Ma, Y.; Chen, L.; Chen, Y. Circulating miR-21 serves as a serum biomarker for hepatocellular carcinoma and correlated with distant metastasis. *Oncotarget* **2017**, *8*, 44050–44058, doi:10.18632/oncotarget.17211.
11. Zhang, Y.; Li, T.; Qiu, Y.; Zhang, T.; Guo, P.; Ma, X.; Wei, Q.; Han, L. Serum microRNA panel for early diagnosis of the onset of hepatocellular carcinoma. *Med. (United States)* **2017**, *96*, doi:10.1097/MD.0000000000005642.
12. Moshiri, F.; Salvi, A.; Gramantieri, L.; Sangiovanni, A.; Guerriero, P.; De Petro, G.; Bassi, C.; Lupini, L.; Sattari, A.; Cheung, D.; et al. Circulating miR-106b-3p, miR-101-3p and miR-1246 as diagnostic biomarkers of hepatocellular carcinoma. *Oncotarget* **2018**, *9*, 15350–15364, doi:10.18632/oncotarget.24601.
13. An, Y.; Gao, S.; Zhao, W.C.; Qiu, B.A.; Xia, N.X.; Zhang, P.J.; Fan, Z.P. Novel serum microRNAs panel on the diagnostic and prognostic implications of hepatocellular carcinoma. *World J. Gastroenterol.* **2018**, *24*, 2596–2604, doi:10.3748/wjg.v24.i24.2596.
14. Han, J.; Li, J.; Qian, Y.; Liu, W.; Liang, J.; Huang, Z.; Wang, S.; Zhao, C. Identification of plasma miR-148a as a noninvasive biomarker for hepatocellular carcinoma. *Clin. Res. Hepatol. Gastroenterol.* **2019**, *43*, 585–593, doi:10.1016/j.clinre.2018.12.008.
15. Weis, A.; Marquart, L.; Calvopina, D.A.; Genz, B.; Ramm, G.A.; Skoien, R. Serum microRNAs as biomarkers in hepatitis C: Preliminary evidence of a microRNA panel for the diagnosis of hepatocellular carcinoma. *Int. J. Mol. Sci.* **2019**, *20*, doi:10.3390/ijms20040864.
16. Yamamoto, Y.; Kondo, S.; Matsuzaki, J.; Esaki, M.; Okusaka, T.; Shimada, K.; Murakami, Y.; Enomoto, M.; Tamori, A.; Kato, K.; et al. Highly Sensitive Circulating MicroRNA Panel for Accurate Detection of Hepatocellular Carcinoma in Patients With Liver Disease. *Hepatol. Commun.* **2020**, *4*, 284–297, doi:10.1002/hep4.1451.
17. Köberle, V.; Kronenberger, B.; Pleli, T.; Trojan, J.; Imelmann, E.; Peveling-Oberhag, J.; Welker, M.W.; Elhendawy, M.; Zeuzem, S.; Piiper, A.; et al. Serum microRNA-1 and microRNA-122 are prognostic markers in patients with hepatocellular carcinoma. *Eur. J. Cancer* **2013**, *49*, 3442–3449, doi:10.1016/j.ejca.2013.06.002.

18. Xu, Y.; Bu, X.; Dai, C.; Shang, C. High serum microRNA-122 level is independently associated with higher overall survival rate in hepatocellular carcinoma patients. *Tumour Biol.* **2015**, *36*, 4773–4776, doi:10.1007/s13277-015-3128-5.
19. Cho, H.J.; Kim, J.K.; Nam, J.S.; Wang, H.J.; Lee, J.H.; Kim, B.W.; Kim, S.S.; Noh, C.K.; Shin, S.J.; Lee, K.M.; et al. High circulating microRNA-122 expression is a poor prognostic marker in patients with hepatitis B virus-related hepatocellular carcinoma who undergo radiofrequency ablation. *Clin. Biochem.* **2015**, *48*, 1073–1078, doi:10.1016/j.clinbiochem.2015.06.019.
20. Cho, H.J.; Kim, S.S.; Nam, J.S.; Kim, J.K.; Lee, J.H.; Kim, B.; Wang, H.J.; Kim, B.W.; Lee, J.D.; Kang, D.Y.; et al. Low levels of circulating microRNA-26a/29a as poor prognostic markers in patients with hepatocellular carcinoma who underwent curative treatment. *Clin. Res. Hepatol. Gastroenterol.* **2017**, *41*, 181–189, doi:10.1016/j.clinre.2016.09.011.
21. Fornari, F.; Pollutri, D.; Patrizi, C.; La Bella, T.; Marinelli, S.; Casadei Gardini, A.; Marisi, G.; Baron Toaldo, M.; Baglioni, M.; Salvatore, V.; et al. In hepatocellular carcinoma miR-221 modulates sorafenib resistance through inhibition of caspase-3–mediated apoptosis. *Clin. Cancer Res.* **2017**, *23*, 3953–3965, doi:10.1158/1078-0432.CCR-16-1464.
22. Nishida, N.; Arizumi, T.; Hagiwara, S.; Ida, H.; Sakurai, T.; Kudo, M. MicroRNAs for the Prediction of Early Response to Sorafenib Treatment in Human Hepatocellular Carcinoma. *Liver cancer* **2017**, *6*, 113–125, doi:10.1159/000449475.
23. Kim, S.S.; Cho, H.J.; Nam, J.S.; Kim, H.J.; Kang, D.R.; Won, J.H.; Kim, J.; Kim, J.K.; Lee, J.H.; Kim, B.H.; et al. Plasma MicroRNA-21, 26a, and 29a-3p as Predictive Markers for Treatment Response Following Transarterial Chemoembolization in Patients with Hepatocellular Carcinoma. *J. Korean Med. Sci.* **2018**, *33*, e6, doi:10.3346/jkms.2018.33.e6.
24. Chuma, M.; Toyoda, H.; Matsuzaki, J.; Saito, Y.; Kumada, T.; Tada, T.; Kaneoka, Y.; Maeda, A.; Yokoo, H.; Ogawa, K.; et al. Circulating microRNA-1246 as a possible biomarker for early tumor recurrence of hepatocellular carcinoma. *Hepatol. Res.* **2019**, *49*, 810–822, doi:10.1111/hepr.13338.
25. Ali, H.E.A.; Emam, A.A.; Zeeneldin, A.A.; Srour, R.; Tabashy, R.; El-Desouky, E.D.; Abd Elmageed, Z.Y.; Abdel-Wahab, A.H.A. Circulating miR-26a, miR-106b, miR-107 and miR-133b stratify hepatocellular carcinoma patients according to their response to transarterial chemoembolization. *Clin. Biochem.* **2019**, *65*, 45–52, doi:10.1016/j.clinbiochem.2019.01.002.
26. Ning, S.; Liu, H.; Gao, B.; Wei, W.; Yang, A.; Li, J.; Zhang, L. MiR-155, miR-96 and miR-99a as potential diagnostic and prognostic tools for the clinical management of hepatocellular carcinoma. *Oncol. Lett.* **2019**, *18*, 3381–3387, doi:10.3892/ol.2019.10606.
27. Jin, Y.; Wong, Y.S.; Goh, B.K.P.; Chan, C.Y.; Cheow, P.C.; Chow, P.K.H.; Lim, T.K.H.; Goh, G.B.B.; Krishnamoorthy, T.L.; Kumar, R.; et al. Circulating microRNAs as Potential Diagnostic and Prognostic Biomarkers in Hepatocellular Carcinoma. *Sci. Rep.* **2019**, *9*, 10464, doi:10.1038/s41598-019-46872-8.
28. Teufel, M.; Seidel, H.; Köchert, K.; Meinhardt, G.; Finn, R.S.; Llovet, J.M.; Bruix, J. Biomarkers Associated With Response to Regorafenib in Patients With Hepatocellular Carcinoma. *Gastroenterology* **2019**, *156*, 1731–1741, doi:10.1053/j.gastro.2019.01.261.
29. Loosen, S.H.; Wirtz, T.H.; Roy, S.; Vucur, M.; Castoldi, M.; Schneider, A.T.; Koppe, C.; Ulmer, T.F.; Roeth, A.A.; Bednarsch, J.; et al. Circulating levels of microRNA193a-5p predict outcome in early stage hepatocellular carcinoma. *PLoS One* **2020**, *15*, doi:10.1371/journal.pone.0239386.
30. Pratedrat, P.; Chuaypen, N.; Nimsamer, P.; Payungporn, S.; Pinjaroen, N.; Sirichindakul, B.; Tangkijvanich, P. Diagnostic and prognostic roles of circulating miRNA-223-3p in hepatitis B virus–related hepatocellular carcinoma. *PLoS One* **2020**, *15*, doi:10.1371/journal.pone.0232211.
